# Comparative Proteomic Analysis of Self‐Compatible *Turnera* Mutants Suggests *Glutathione S‐Transferase 8* Is Involved in Overcoming S‐Morph Female Self‐Incompatibility Response

**DOI:** 10.1002/pld3.70125

**Published:** 2025-11-28

**Authors:** Paige M. Henning, Paul D. J. Chafe, Hasan J. Hamam, Joel S. Shore

**Affiliations:** ^1^ Center for Genomic Science Innovation University of Wisconsin‐Madison Madison Wisconsin USA; ^2^ Department of Biology York University Toronto Ontario Canada

**Keywords:** distyly, glutathione S‐transferase, mating type, pollen, ROS signaling, self‐incompatibility

## Abstract

Distyly is a reproductive system, characterized by the presence of two floral morphs, which promotes outcrossing via physical and biochemical means. In distylous *Turnera*, the mating type of the S‐morph is determined by two genes: *YUC6* (male) and *BAHD* (female). Despite the importance of these *S*‐genes, it is likely that additional genes are involved in the distylous syndrome. Here, we use comparative mass spectrometry analysis to identify differentially expressed proteins in a series of self‐compatible mutants and wildtype distylous members of *Turnera*. Our analysis identified a member of the *Glutathione S‐transferase* family that overwhelmingly correlated with L‐morph male mating type. Exploration of the large datasets and previously published work led to the proposal that differential ROS levels in the pistil may contribute towards the self‐incompatibility response. To support this hypothesis, we generated a co‐expression network for whole flower buds from self‐compatible and WT *Turnera joelii*. This network led to the identification of a series of ROS and auxin‐related genes that correlated with self‐compatibility. We update previously proposed SI response models to reflect how ROS, jasmonic acid, and brassinosteroid signaling likely establish the S‐morph female self‐incompatibility response. Overall, this work has identified genes potentially related to self‐compatibility and has provided a foundation for future empirical work investigating the basis of the SI response in *Turnera*.

## 1 Introduction

**FIGURE 1:**
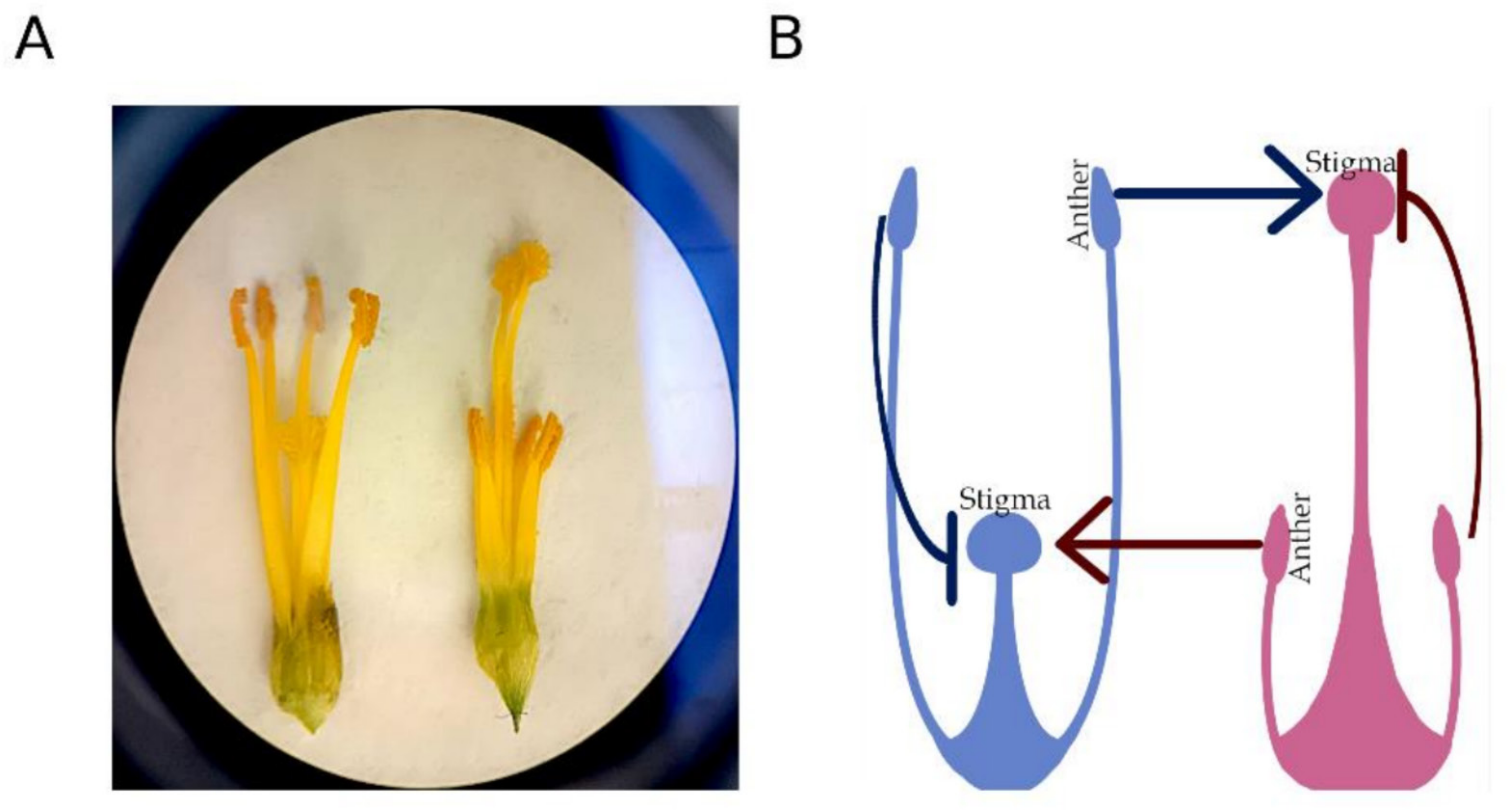
Image of the S‐morph (left) and L‐morph (right) of *T. joelii* flowers with corolla removed (A). Diagram depicting compatibility relationships in crosses between an S‐ and L‐morph individual (B). Compatible crosses are denoted with an arrow, and incompatible crosses are denoted with a line. Color denotes the origin of pollen (blue S‐morph; pink L‐morph).

Distyly, a form of heterostyly, has been a system of interest for over a century, with early research dating to the 1800s (Darwin 1877). Interest in the system stems from the presence of self‐ and intra‐morph incompatibility coupled with reciprocal herkogamy (Figure 1A,B), a unique form of herkogamy in which one floral morph, the L‐morph (Long‐styled) exhibits approach herkogamy (stigma above anthers), while the S‐morph (Short‐styled) exhibits reverse herkogamy in which the stigma resides below the anthers. The phenotypes associated with distyly are highly conserved across 28 families, making it an exceptional case of convergent evolution and of interest to a variety of fields (Naiki 2012; Barrett 2019).

The basis of distyly in all genera studied to date is the *S*‐locus, a hemizygous supergene composed of two or more *S*‐genes that confers the S‐morph phenotype (Nowak et al. 2015; Shore et al. 2019; Gutiérrez‐Valencia et al. 2022; Zhao et al. 2023; Fawcett et al. 2023; Yang et al. 2023; Raimondeau et al. 2024). Absence of the *S*‐locus in the genome results in the L‐morph, indicating that the L‐morph is the “default” morph in distylous systems.

Despite the importance of the *S*‐genes, their presence/absence alone likely cannot explain all aspects of the distylous syndrome, but rather the *S*‐genes may act as master regulators of other gene(s)/protein(s). This hypothesis has been supported by empirical evidence and by the hypothetical roles of the *S*‐genes from many genera. The strongest evidence is that of the *S*‐genes from *Primula*, *Turnera*, and *Forsythia suspensa* . Negative regulation of brassinosteroids (BR) levels in *Primula* (Huu et al. 2020, 2021), *Turnera* (Matzke et al. 2020, 2021), and *F. suspensa* (Song et al. 2024) represses style elongation in the S‐morph as determined using transgenic lines and exogenous BR treatments and, in the case of *Primula* and *Turnera*, establishes female mating type. In *Turnera*, upregulation of auxin levels in the S‐morph's anthers establishes male mating type and pollen size dimorphisms, as determined with transgenic lines (Henning et al. 2022). In *Primula*, *GLO* ^*T*^, a MADS‐BOX gene, encourages cell elongation in the corolla to establish anther height dimorphisms as determined by knocking down *GLO* ^*T*^ expression (Huu et al. 2020).

In addition to the empirical evidence from *Forsythia*, *Primula*, and *Turnera*, the hypothetical roles of *S*‐genes in other genera are overwhelmingly related to the regulation of phytohormones (Zhao et al. 2023; Yang et al. 2023; Raimondeau et al. 2024), and one *S*‐gene from *Turnera* encodes a peptide hormone (Shore et al. 2019). Overall, this suggests the *S*‐genes from a variety of genera regulate phytohormone levels to establish the distylous syndrome.

Aside from regulating hormone levels, some *S*‐genes directly regulate transcription by acting as transcription factors, i.e., *GLO* ^*T*^ from the *Primula* system (Huu et al. 2020) and *S‐ELF3* from the *Fagopyrum* system (Yasui et al. 2012). Taken together, it is likely that the *S*‐genes act as master regulators of genes not linked to or outside of the *S*‐locus. Thus, hypothetically, genes outside of the *S*‐locus are just as crucial for the distylous syndrome as the *S*‐genes.

The *S*‐genes' roles as master regulators have been demonstrated numerous times using RNA‐seq (Cohen 2016; Burrows and McCubbin 2018; Zhao et al. 2019; Henning et al. 2020; Potente et al. 2022; Song et al. 2024; Lin et al. 2025), differential DNA‐methylation analysis (Sala‐Cholewa et al. 2024), phosphoproteomics (Henning et al. 2024), and proteomics (Khosravi et al. 2004; Kalinowski et al. 2007; Ushijima et al. 2012; Henning et al. 2024). The importance of unlinked genes has also recently been empirically supported in *Primula*, as the unlinked gene *PIN* is negatively regulated by the *S*‐gene *CYP* ^*T*^, resulting in the S‐morph's short style phenotype (Liu et al. 2024).

In *Turnera*, the *S*‐locus is composed of three *S*‐genes: *YUC6*, a flavin monooxygenase, which establishes male mating type by increasing IAA concentrations; *SPH1*, a peptide hormone, which promotes filament elongation in the S‐morph; and *BAHD*, an acyltransferase, which represses style elongation and establishes female mating type via inactivation of BR (Shore et al. 2019). Previously, we proposed that alteration of IAA and BR levels is responsible for establishing the general S‐morph mating type (Henning et al. 2020).

Ectopic expression of *BAHD* in *Arabidopsis thaliana* results in altered expression of notable BR‐responsive genes (Matzke et al. 2020), supporting BAHD's role in BR inactivation. However, exogenous treatment of *Turnera joelii* pollen with BR did not result in a morph‐specific SI response (Matzke et al. 2021), suggesting BR itself does not confer S‐morph female mating type, but BR regulated genes likely are involved in establishing female mating type. In support of BAHD's role as a master regulator through BR inactivation, two genes, an α‐dioxygenase (Khosravi et al. 2004) and AT‐hook‐like protein (AHL; Henning et al. 2020), have been identified as potential key players in establishing the female aspects of distyly. Some members of the AHL family are regulated by BR (Favero et al. 2020), and α‐dioxygenase is regulated by jasmonate (Hong et al. 2017), which has an antagonistic relationship with BR (Saini et al. 2015).

Similar alterations of the expression of IAA‐responsive genes were observed when knocking down the expression of the *S*‐gene *TjYUC6* in *T. joelii* (Henning et al. 2022). One knockdown line exhibited an L‐morph male mating type (henceforth L‐male mating type), and IAA‐responsive genes in that line showed expression patterns comparable to the WT L‐morph. This analysis also identified the presence of *YUC6* transcript in S‐morph desiccated pollen. It is currently unknown if IAA directly establishes S‐morph male matingtype (henceforth S‐male mating type) or if altered expression of auxin‐regulated genes establishes S‐male mating type.

The presence of *YUC6* transcript in desiccated pollen in conjunction with the S‐morph's pollen genotypes (only half of the pollen contains the *S*‐locus), suggests that developmental priming, a phenomenon in which transcripts and proteins are stored in pollen for later rapid response to stigmatic cues or stressors (Chaturvedi et al. 2016), may be involved in the establishment of the S‐male mating type. If this is the case, IAA production during germination or pollen tube elongation may be important for establishing the S‐male mating type, the observed pollen tube dimorphisms (rapid in vitro growth of the S‐morph's tube relative to the L‐morph's), and/or the previously observed differences in male SI response (Safavian and Shore 2010).

To date, there has not been an exploration of the potential role of reactive oxygen species (ROS) in heteromorphic SI, likely because all identified *S*‐genes to date are completely unrelated to ROS. Additionally, none of the RNA‐seq analyses strongly suggested ROS may play a role in heteromorphic SI. However, as ROS is involved in some forms of unrelated homomorphic SI (Wilkins et al. 2011; Zhang et al. 2021), it remains a possibility that ROS may be involved in heteromorphic SI. In homomorphic SI systems, the perception of a self‐ligand by a self‐receptor results in increased ROS levels in the stigma (Zhang et al. 2021) or pollen tube (Wilkins et al. 2011) triggering a programmed cell death (PCD) response. Exploring the potential role of ROS in heteromorphic SI may be necessary, as pollen tube death in *Turnera* may be a result of PCD (Safavian and Shore 2010).

Exploration of the transcriptomes and proteomes of pollen can help support hypotheses regarding the potential role of developmental priming in male mating type and/or aid in forming hypotheses regarding which proteins are important for these dimorphisms and male SI response. As such, a proteomic analysis of the pollen from SC mutants and WT S‐morph individuals may yield some insight into why *YUC6* transcript is present in mature pollen.

Here, we present the first proteomic analysis of pollen for any distylous species. Using a series of self‐compatible (SC) mutants, we form hypotheses regarding the potential involvement of ROS‐mediated signaling in establishing SI response and the potential importance of developmental priming. Overall, this work has identified a series of proteins hypothetically important for male SI response, forming an important foundation for future empirical work.

## 2 Results

### 2.1 Mutations Outside of the *S*‐Locus Can Result in Self‐Compatibility

**FIGURE 2:**
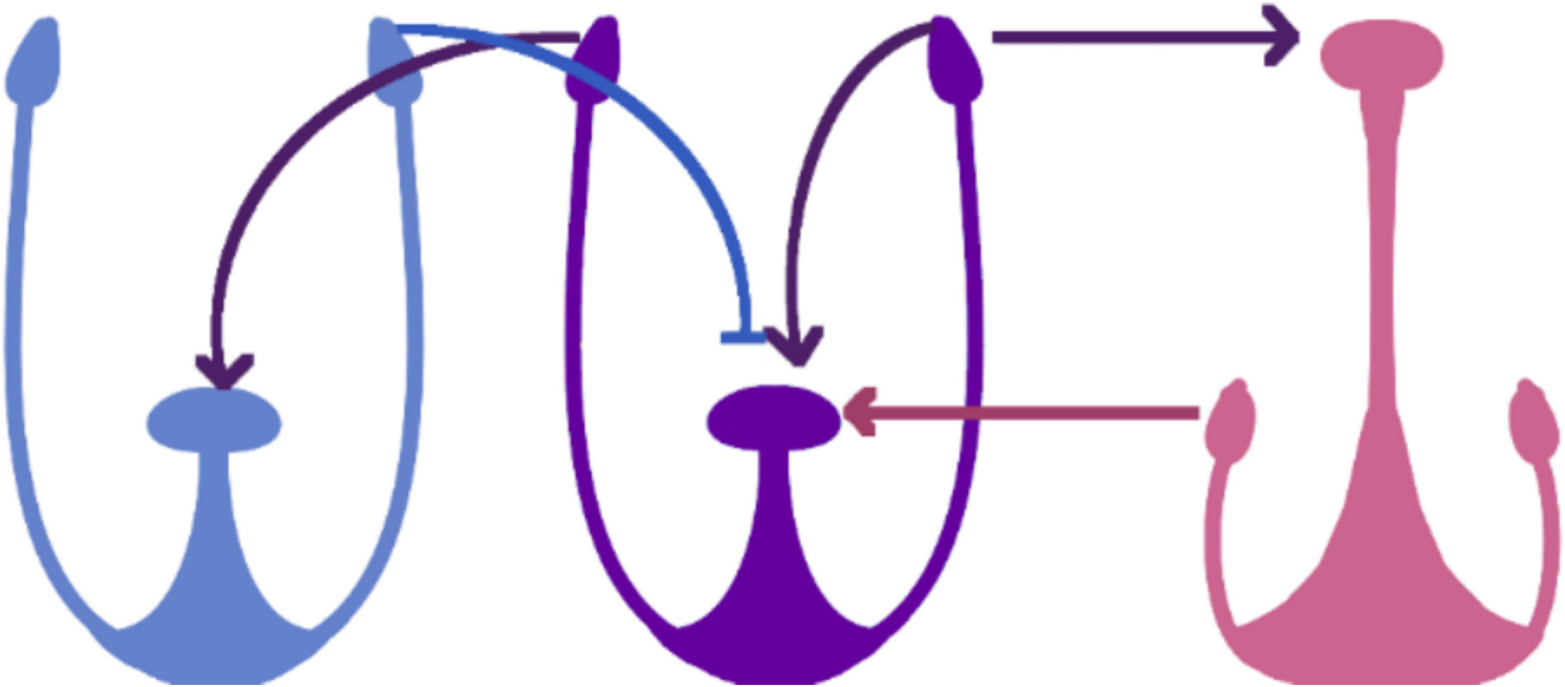
Model of crosses regarding self‐compatible S‐morph mutant (center), relative to WT S‐morph (left), and WT L‐morph (right). Purple = mutant, blue = WT S‐morph, and pink = WT L‐morph. Arrows denote compatible/successful cross. Lines denote incompatible/unsuccessful cross. Color denotes origin of pollen.

Three SC mutants, previously identified or generated at York University, were used in this analysis. In all cases, the mutants originated from an S‐morph individual. SC mutants all exhibited an altered male mating type, which should be considered “null” as their pollen is capable of fertilizing both WT S‐ and L‐morph individuals, which is atypical of distylous members of *Turnera* (Figure 2). In all cases, the SC mutants exhibited a WT S‐morph female mating type as only pollen from WT L‐morph individuals or self‐pollen could fertilize the SC mutant.

#### 2.1.1 SC *Turnera scabra* Mutant

*T. scabra* from El Salvador and Managua Nicaragua were crossed to produce ESMAN, an S‐morph diploid individual. ESMAN was subjected to X‐ray mutagenesis during a pilot study to identify mutations associated with distyly. One shoot on the plant became SC. This shoot was subsequently separated from ESMAN and both mutant and nonmutant (WT) shoots of the plant were propagated via cuttings for numerous years and retained their phenotypes.

Pollen from the SC mutant is capable of fertilizing both S‐ and L‐morphs, but the mutant is only capable of setting seed when receiving pollen from self or L‐morph plants. Selfing the mutant resulted in 4 S‐morph:39 L‐morph progeny, an extreme departure from the expected 3 S‐morph:1 L‐morph ratio (Table 1).

**TABLE 1:** Biased transmission of the S‐morph in *T. scabra* and *T. joelii* mutants.

Plant	Form of crossing	Expected ratio	Progeny		Χ^2^df = 1	*p*
			S‐morph	L‐morph		
***T. scabra***						
ESMAN	SC selfed	3S:1L	4	39	98.9	< 0.0001
***T. joelii* parental line** ^a^						
J30L × SI	WT L × WT S	1S:1L	13	11	0.167	n.s.
SC × J30L	SC × WT L	1S:1L	1	28	79.184	< 0.0001
J211S × SC^b^	WT S × SC	3S:1L	10	11	8.39	0.0038
SC	SC selfed	3S:1L	3	28	70.55	< 0.0001
***T. joelii* generation 1**						
SC × J30L‐12S	Selfed	3S:1L	6	10	12	0.0005
SSC‐4S	Selfed	3S:1L	4	8	11.11	0.0009
SSC‐13S	Selfed	3S:1L	6	31	68.18	< 0.0001
SSC‐18S	Selfed	3S:1L	2	4	5.556	0.0184
***T. joelii* generation 2**						
SC‐J30L‐12S	Selfed	3S:1L	41	53	49.38	< 0.0001
SSC‐4S	Selfed	3S:1L	9	64	152.92	< 0.0001
SSC‐13S	Selfed	3S:1L	10	24	37.69	< 0.0001
SSC‐18S	Selfed	3S:1L	4	19	40.710	< 0.0001

Abbreviation: n.s., not significant.

^a^ Female parent × male parent.

^b^ All but one of the S‐morph progeny from this cross was self‐incompatible with 1 plant setting a mean of 0.5 seeds.

Three of the S‐morph progeny were selfed to explore the inheritance of SC; only one of the progeny produced any seed at all, and at a much lower degree than the parental mutant (mean of 0.3 ± 0.1 seeds). While the genetic basis of SC is unknown, mutation(s) of the *S*‐genes is unlikely, as the three S‐morph progeny did not exhibit self‐compatibility, but must have inherited the *S*‐locus from the SC plant. In addition to the extreme transmission bias against the *S*‐genes, the gene(s) conferring SC, did not appear to be transmitted to the S‐morph progeny, although sample sizes are small.

#### 2.1.2 SC *T. joelii* S‐Morph Mutant

An S‐morph plant of *T*. joelii spontaneously gained an apical somatic mutation that resulted in the upper shoots of the plant becoming SC (Figure S1). Cuttings of SI and SC shoots were rooted and propagated for many years, retaining their phenotypes.

**FIGURE 3:**
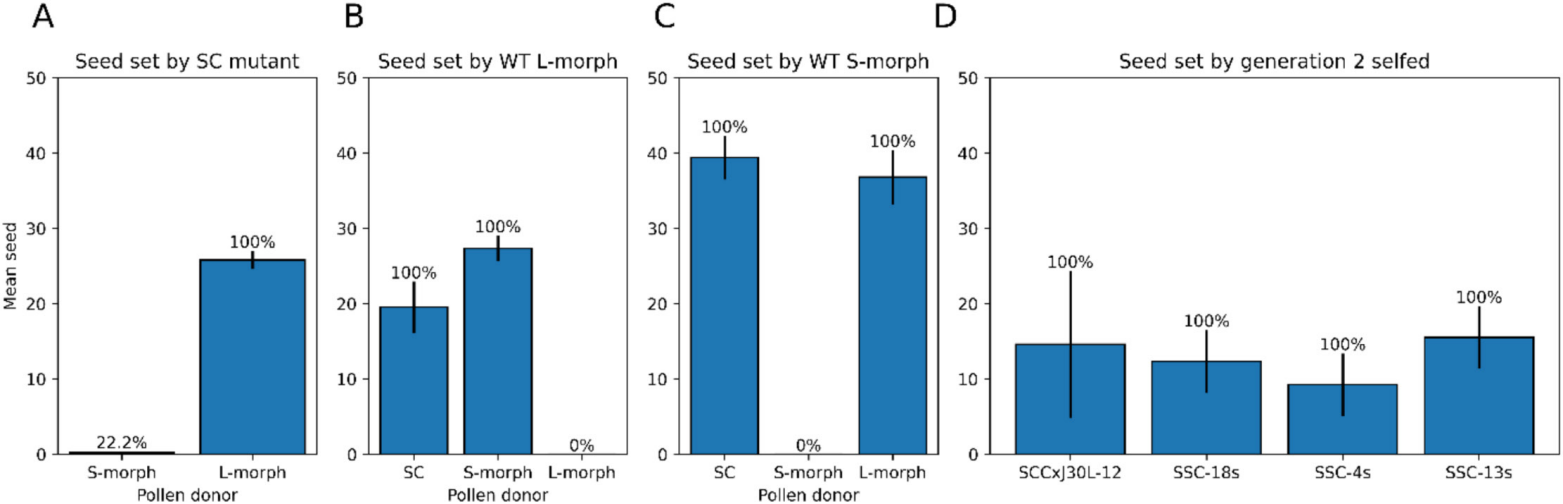
Mean seed set for generation 1 of the SC *T. joelii* mutant relative to WT S‐ and L‐morph *T. joelli* (A–C). SC mutant is acting as the female plant/pollen receiver (A). WT L‐morph is acting as the female plant/pollen receiver (B).WT S‐morph is acting as the female plant/pollen receiver (C). Percentages above bars represent the percent fruit set. *n* = 7–9 (A–C). Mean seed set for generation 3 of SC mutant lineage when progeny is selfed; *n* = 10 (D). Error bars represent the standard error on all graphs.

While the SC mutant's female mating type remains S (Figure 3A), the male mating type has changed (Figure 3B,C). The mutant appears to be “null” with respect to pollen mating type as it can fertilize either morph. It is likely that the small amount of seed set in the S‐morph × SC mutant cross (mean = 0.22 ± 0.15 seed) was a result of self‐pollen contamination, as the mutant's flowers were not emasculated prior to anthesis.

Similar to the *T. scabra* ESMAN mutant, crosses involving the SC *T. joelii* mutant resulted in a large bias against transmission of the *S*‐locus (Table 1). This trend was observed across three generations (Table 1). The cause of the transmission bias is unknown.

None of the L‐morph progeny from the lineage were SC, while all S‐morph progeny were SC. The extent of selfed seed set varied in some later‐generation progeny (Figure 3D), with some third‐generation S‐morph offspring producing only a mean 2.25 ± 4.09 seeds (*n* = 10) when selfed. We do not know if the reduced seed set is a function of a reduced degree of self‐compatibility or a result of inbreeding depression. As the mutants maintained a WT genomic copy of *YUC6* (Figure S2) and all *S*‐genes were expressed (Data S1; Figure S3), the mutation results in SC is not in the *S*‐locus. However, as the mutation is inherited with the *S*‐locus, it is likely that the mutated gene(s) are tightly linked to the *S*‐locus.

### 2.2 SC *T. joelii* Mutants Have Significantly Smaller Pollen Size Than WT Counterparts

A second SC *T. joelii* line (T1S2) was spontaneously generated during the development of the *T. joelii* transformation process (Chafe et al. 2015). T1S2's offspring showed similar departure from the expected 3S:1L inheritance and its pollen was significantly smaller than WT S‐morph (Chafe et al. 2015). Similar to T1S2, knockdown *TjYUC6* lines (KD) showed varied pollen phenotypes that correlated to the degree of *YUC6* expression (Henning et al. 2022). These previous results suggest that S‐male mating type and pollen size dimorphisms are linked to some degree.

**FIGURE 4:**
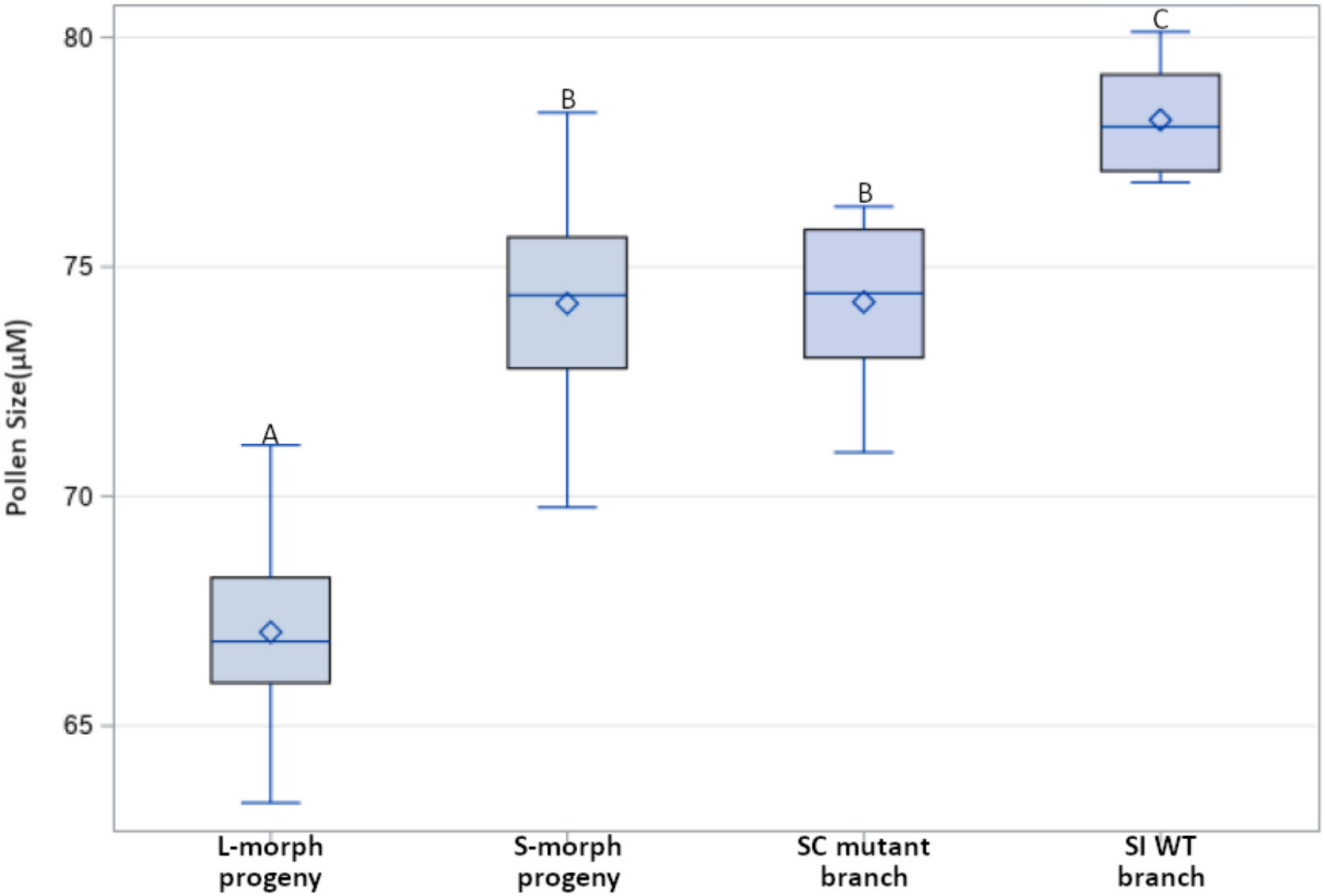
Box and whisker plots of pollen size from the SC and SI branches of the original *T. joelii* plant and pollen size of the SC branch's S‐ and L‐morph progeny. Statistical significance determined by one‐way ANOVA and Tukey's HSD; alpha = 0.05, degrees of freedom = 200.

To explore this hypothesis, we measured the pollen grains of the SC *T. joelii* line described above and its progeny. Pollen from the original SC branch and its S‐morph progeny was significantly different from the WT S‐morph and the SC branch's L‐morph progeny (Figure 4). Overall, this suggests that the male mating type and pollen size dimorphisms genetic networks are shared to some degree beyond *YUC6*. Considering the number of auxin‐responsive proteins that were identified as downregulated in the SC relative to the WT S‐morph (see discussion below), this result supports previous hypotheses that decreased auxin levels negatively affect pollen size in the S‐morph.

### 2.3 MS Analysis of SC *T. scabra* Mutant Identifies Proteins Involved in Auxin Storage and Homeostasis

Mass spectrometry (MS) analysis of the pollen from the SC and WT shoots of ESMAN identified 9772 unique peptides from 1287 proteins (Data S2). Two proteins were only identified in the WT samples and not in the SC mutant (henceforth these are called WT‐specific): a cystatin domain‐containing protein (CDCP, with homology to Tsubulata_005748/KAJ4845612.1) and an ILR1‐like1 protein (ILL1, with homology to Tsubulata_033406/KAJ4845591.1). The WT specificity of these proteins suggests they are not expressed in the SC mutant or are expressed at levels below our limits of detection. Given that X‐rays can generate deletions, it is possible that functional copies of the genes are not present in the mutant's genome. None of the other proteins detected were significantly differentially expressed (DE) between the mutant and WT plants.

### 2.4 MS Analysis of an SC *T. joelii* Mutant Identifies Proteins Involved in ROS Signaling and Homeostasis

MS analysis of the pollen from the SC vs. WT shoots of the *T. joelii* mutant identified 13,621 unique peptides from 1658 proteins (Data S2). Of these, 33 were DE (foldchange [FC] ≤ 0.5 or FC ≥ 1.5, *q*‐value < 0.05), 10 were SC mutant specific, and five were WT‐specific.

To further filter the results, two pollen samples from an SC offspring of the mutant plant were included in a second analysis to test the consistency of the results. MS analysis (SCT_1_&SCT_2_vsWT) identified 14,907 unique peptides from 1697 proteins (Data S2). Of the identified SCT_1_ specific proteins, only one was found in both generations to be SC‐specific, a putative Purple Acid Phosphatase (PAP/Tsubulata_027257/KAJ4849921.1) family member.

Twenty‐five of the DE proteins were shared between the SCT_1_vsWT and SCT_1_&SCT_2_vsWT analyses (Table S1). The protein with the lowest expression in both generations relative to the WT was SBT5 (Tsubulata_033680/KAJ4847151.1) SBT5 is a *Subtilisin‐like Serine Protease* whose closest *Arabidopsis* homolog, AT2G04160, is upregulated in response to auxin (Neuteboom et al. 1999).

Similar to the SC *T. scabra* , two cystatin family members (Tsubulata_040299/KAJ4851318.1 and Tsubulata_001228/KAJ4825724.1) were upregulated in the SC *T. joelii* relative to the WT plant (Table S1). Both cystatins are classified as PhyCys‐I cystatins based on their predicted molecular weights (Balbinott and Margis 2022).

### 2.5 Differential Analysis of WT S‐ vs. L‐Morphs of *T. joelii* and *Turnera subulata* Identify a *Glutathione S‐Transferase* (GST) That May Aid in Establishing L‐Male Mating Type

MS analysis of the WT S‐ vs. L‐morphs of distylous *T. joelii* and *T. subulata* was performed. As distyly is an ancestral trait in *Turnera*, the two species were treated as one in our SvL analysis to remove any differential expression unrelated to distyly; this method was previously utilized in an RNA‐seq analysis (Henning et al. 2020). MS analysis captured 24,170 unique peptides from 2371 proteins (Data S2); of these, 20 were DE (SvL), 24 were only identified in the S‐morph, and 13 were only identified in the L‐morph. Despite the previous identification of *YUC6* transcript in the pollen of the S‐morph (Henning et al. 2022), YUC6 peptides were not identified, suggesting that YUC6 is either below our levels of detection or is not translated until pollen germination.

The differentially expressed proteins (DEP) from all MS analyses were compared to quickly identify protein(s) of interest (Figure S4). GST8 (Tsubulata_039799/KAJ4828672.2), a putative member of the *GST* family, was the sole protein shared between three of the four analyses. *GST8* was formerly part of Tsubulata_039797 (KAJ4828671.2); however, manual curation found Tsubulata_039797 was composed of 11 *GST* family members. In *T. subulata* and *T. joelii*, GST8 is the second most upregulated protein in the L‐morph, suggesting it may be important for establishing L‐male mating type. Furthermore, GST8 showed higher expression in the SC mutant *T. joelii* relative to WT (Table S1), suggesting that the ability of the SC mutant to fertilize S‐morph plants may be due to the upregulation of GST8.

To further support a hypothetical role for GST8 in establishing L‐male mating type, we performed MS analysis for a second SC *T. joelii* mutant, referred to as “T1S2,” that was spontaneously generated during the development of the *T. joelii* transformation method (Chafe et al. 2015). Similar to the *T. joelii* SC mutant above, T1S2 also exhibits “null” pollen response and can fertilize both L‐ and S‐morphs while acting as a pollen parent. T1S2's stigma rejects wild type S‐morph pollen, but it can be pollinated by L‐morph pollen. The genetic basis of SC in T1S2 is unknown. MS analysis identified 15,487 peptides from 1719 proteins (Data S2). Due to low levels of fertility of the T1S2 mutant and the smaller replicate number (*n* = 2), the dataset will be used only as additional support. GST8 was upregulated in the mutant, supporting GST8's hypothetical importance in establishing L‐male mating type.

### 2.6 Five Gene Families May Be Key Players in Establishing Male Mating Type or Pollen Dimorphisms

To further identify potential key players in establishing male mating type, DEP were assigned to gene families based on homology; these were filtered to identify shared DEP families of potential interest. Five gene families contained members that were DE between treatments (Table 2).

**TABLE 2:** Differentially expressed gene family members.

Family	WT *T. subulata* and *T. joelii* (SvL)		SC *T. joelii* (SC_T1_&SC_T2_vWT)		ESMAN SC *T. scabra* (SCvWT)		T1S2 SC *T. joelii* (SCvWT)	
*Cystatin*	Tsubulata_002471 KAJ4827122.1	FC = 0.055 *q* = 0.0140	Tsubulata_040299 KAJ4851318.1	FC = 75.173 *q* = 0.0087	Tsubulata_005748 KAJ4845612.1	FC = 0.001		
			Tsubulata_001228 KAJ4825724.1	FC = 54.128 *q* = 0.03147				
*IAA‐amino hydrolase family*					Tsubulata_033406 KAJ4845591.1	FC = 0.001	Tsub_00016677 KAJ4823680.1	FC = 0.001
*NAD(P)‐binding Rossmann‐fold*	Tsubulata_001814 KAJ4842460.1	FC = 23.4 *q* = 0.0022	Tsubulata_026328 KAJ4824385.1	FC = 0.362 *q* = 0.01025			Tsubulata_002790 KAJ4835277.1	FC = 0.001
	Tsubulata_008166^a^ KAJ4850237.1	FC = 1000	Tsubulata_002790 KAJ4835277.1	FC = 0.166 *q* = 1.62E‐06			Tsubulata_039467 KAJ4822791.1	FC = 0.001
							Tsubulata_033831 KAJ4850850.1	FC = 0.001
							Tsubulata_036035 KAJ4821943.1	FC = 0.001
							Tsubulata_008166 KAJ4850237.1	FC = 0.001
							Tsubulata_028667 KAJ4840870.1	FC = 0.001
*UDP‐glycosyltransferase*	Tsubulata_007478 KAJ4829682.1	FC = 2.842 *q* = 0.014	Tsubulata_002930 KAJ4840182.1	FC = 190.88 *q* = 0.0028			Tsubulata_046794 KAJ4846253.1	FC = 2.975 *q* = 0.0453
	Tsubulata_044155 KAJ4847074.1	FC = 53.45 *q* = 1.40E‐05						
*Glutathione S‐transferase*	Tsubulata_039799 KAJ4828672.2	FC = 0.097 *q* = 0.0365	Tsubulata_039799 KAJ4828672.2	FC = 8.284 *q* = 0.0183			Tsubulata_039799 KAJ4828672.2	FC = 24.822 *q* = 0.036251

^a^ Does not contain tropinone reductase motif.

Another class PhyCys‐I cystatin (Tsubulata_002471/KAJ4827122.1) was identified as upregulated in the WT L‐morph relative to the S‐morph.

A second member of the *IAA‐amino acid hydrolase* family (*ILL1‐4*, Tsubulata_027681, KAJ4823680.1) was identified as WT‐specific in the T1S2 dataset. *ILL1‐4* was also upregulated (FC = 0.321) in WT *T. joelii* relative to the spontaneous somatic mutant; however, this difference was not statistically significant (*q* = 0.691). *ILL1‐4* was identified as not DE in the SvL comparison (FC = 0.702).

Exploration of DEP associated families also identified the potential importance of *NAD(P)‐binding Rossmann‐fold* (NBRF) proteins in establishing S‐male mating type, as two family members are upregulated in the WT S‐morphs used in our SvL and SCvWT analyses (Table 2). All proteins contain a short‐chain dehydrogenase/reductase (IPR002347) domain, a glucose/ribitol dehydrogenase (PR00081) signature, and three of the proteins contain a tropinone reductase (IPR045000) motif. Exploration of the T1S2 dataset further supported the hypothetical importance of NBRF members, as peptides from six members were not identified in the T1S2 mutant (Table 2).

### 2.7 Co‐Expression Analysis of *T. joelii* SC Mature Buds Supports the Role of GST8 in Self‐Incompatibility (SI) Response

To identify transcriptional changes associated with the SC phenotype, we generated a co‐expression network using previously published RNA‐seq data from the WT S‐ and L‐morph (Henning et al. 2020) and unpublished data from the SC *T. joelii* mutant collected during the same period. Considering the RNA‐seq of the mutant was collected at York University, while the RNA‐seq for WT plants was collected at Washington State University, the buds likely represent slightly different stages of development, as suggested by the expression profiles of the *S*‐genes (Figure S3). With this in consideration, we focused our analysis on the mature buds, as the SC mutant showed comparable levels of *YUC6* expression to the mature buds. After filtering, WGNCA identified eight modules that significantly correlated with the distylous syndrome or with the SC mutant (Figure S5 and Tables S2 and S3). Modules represent groups of genes that are likely involved in the same biological function. Modules have been arbitrarily named different colors as a default setting of WGNCA.

Of the statistically significant modules (8/16), three correlated with self‐compatibility (magenta and turquoise positively correlated and cyan negatively correlated). *GST8* was associated with the turquoise module, which positively correlated with SC (Table S4). *S*‐genes *SPH1* and *BAHD* were found in the pink module, while *YUC6* was associated with the blue module.

*BAHD* and *SPH1* were associated with the pink module, which has the second strongest correlation to the S‐morph; the module's positive correlation with “S‐morph male SI” is likely reflective of the low expression values of *BAHD* and *SPH1* (Figure S3B,D), likely due to differences in collection age rather than a reflection of mutated expression patterns of the *S*‐genes themselves. A previously identified style‐specific α‐dioxygenase (α‐DOX^S^/Tsubulata_027817; Khosravi et al. 2004) and AT‐hook motif nuclear‐localized protein were associated with this module (Tsubulata_035210; Henning et al. 2020). Several previously identified DE genes, DEP, and differentially phosphorylated sites were also associated with this module. GO term analysis of the module's closest *Arabidopsis thaliana* homologs revealed that the dataset is enriched with genes annotated as peroxisomes (GO:0005777; Figure S6). Response to light (GO:0009416) was also enriched, supporting a previous hypothesis that phytochrome interacting factor signaling hubs are involved in the repression of style elongation (Henning et al. 2020). Several GO terms related to post‐translational modifications, signaling, and cell differentiation were also enriched in the module, potentially supporting previous hypotheses regarding SPH1's role in regulating cell division (Henning et al. 2024).

*YUC6* was associated with the blue module; this module showed the highest correlation to the S‐morph. To explore the function of this module, we analyzed enriched GO terms and KEGG pathways for the closest *A. thaliana* homologs (Figure S7). The most enriched KEGG pathways were “one carbon pool by folate” (ath00670) and “pyruvate metabolism” pathway (ath00620). Of the 15 enriched KEGG pathways associated with the blue module, four were linked to transcription, one was linked to post‐transcriptional modifications, and one was linked to translation.

To identify the basis of SC/null mating type, we examined the modules that correlated with SC; magenta and turquoise were positively correlated with SC, and cyan was negatively correlated with SC. The magenta and turquoise modules formed one larger module (Figure S8). *GST8* is part of the turquoise module and showed expression similar to genes in the magenta and turquoise modules, as determined by first neighbors; interactions between the two modules suggest they are both important for establishing SC. Surprisingly, seven additional *GST* family members were associated with the turquoise module (Table S4). One, *GST7* (Tsubulata_027213, KAJ4837327), was identified in the MS dataset, but was not DE (FC = 1.044). The remaining members were not identified in the 2020 analysis or our pollen analysis.

Forty‐four previously identified DE genes from the mature stamen of *T. subulata* and *T. joelii* were associated with the magenta and turquoise modules, and 35 formed a subnetwork (Figure S9; Henning et al. 2020). Of particular interest was *GLUTATHIONE PEROXIDASE 6* (GPX6, Tsubulata_004501, KAJ4823232), which was previously identified as downregulated in the S‐morph's mature stamen relative to the L‐morph's. GPX6 had a 1.58 foldchange (*q*‐value = 0.021) in our comparison of mRNA levels in the SC and WT L vs. WT S and an insignificant fold change of 2.85 (*q*‐value = 0.371) when comparing SC to WT L and S; this insignificance may be a result of low replicate number (*n* = 2) for the SC mutant. Based on these expression patterns, GPX6 may be of interest for future work. Two additional GPX family members (Tsubulata_004502 and Tsubulata_009340) and a *Nitrilase‐like protein* family member (*NIT1*, Tsubulata_010911, KAJ4824598.1) were also identified in the turquoise module.

Three auxin‐related genes, *IAA14* (Tsubulata_012549, KAJ484623), a methyl IAA esterase (*MES17*, Tsubulata_007335, KAJ4834393), and a cytochrome P450 (*CYP79B3*, Tsubulata_043398, KAJ4824572.1) also correlated with SC.

To identify pathways associated with the mutation, we explored enriched KEGG pathways in the magenta and turquoise modules and the negatively correlated cyan module. The magenta and turquoise contained 13 enriched pathways (Figure S10). Of these, three were related to DNA repair, two were related to DNA synthesis, two were related to mRNA degradation, and two were related to protein synthesis, potentially alluding to genome instability in the SC mutant. Two KEGG pathways were enriched in the cyan module: tryptophan metabolism (ath00380) and MAPK signaling (ath04016).

## 3 Discussion

In distylous *Turnera*, two *S*‐genes are responsible for establishing SI in the S‐morph: *YUC6*, which establishes male SI via auxin production (IAA; Henning et al. 2022), and *BAHD*, which establishes female SI via inactivation of BR (Matzke et al. 2021). As IAA and BR are both heavily used in signaling, it is unlikely that the *S*‐genes themselves confer SI, but rather altered levels of IAA and BR result in transcriptional, translational, and post‐translational differences between the S‐ and L‐morphs.

It is evident this is the case with *BAHD*, as treating elongating pollen tubes with different BR derivatives does not result in a morph‐specific response (Matzke et al. 2021). Furthermore ectopic expression of *BAHD* in *A. thaliana* altered the expression of BR‐responsive genes (Matzke et al. 2020). This hypothesis has, in part, also been supported by *YUC6* knockdown lines of *T. joelii* (Henning et al. 2022). Decreases in *YUC6* expression resulted in changes in the expression of IAA‐responsive genes, reflecting expression patterns statistically similar to that of the L‐morph. Additionally, transcriptomic (Henning et al. 2020) and proteomic (Khosravi et al. 2004; Henning et al. 2024) studies have shown statistical differences between the transcriptomes, proteomes, and phosphoproteomes of the S‐ and L‐morphs' developing buds.

While these transcriptomic and proteomic analyses have identified genes/proteins that are potential key players in establishing female SI response and reverse herkogamy, they have failed to identify a strongly supported potential key player(s) in male SI responses. To date, ‐omic analyses have only identified auxin‐responsive genes that may be manipulated in response to differential IAA levels. As proteomic analysis of the pollen of tristylous *Lythrum salicaria* identified several DE pollen proteins between the three floral morphs (Kalinowski et al. 2007), exploration of the *Turnera* pollen proteome may provide insights into male SI response.

Here, we used comparative proteomics to analyze the potential involvement of various proteins in the SI response observed in distylous *Turnera*. Analysis of a series of SC S‐morph mutants and WT S‐ and L‐morph plants revealed a potential role for ROS signaling in the SI response in the S‐morph of *Turnera*, pointing towards two key players in the SI response: a pollen‐expressed GST and a previously identified S‐morph‐specific stigma‐expressed α‐dioxygenase. To support this hypothesis, we generated a co‐expression network of the mature buds of SC *T. joelii* and WT *T. joelii*. In doing so, we provide additional evidence for the importance of auxin biosynthesis in S‐male mating type, support the proposed importance of ROS in the SI response, support the proposed role of *GST8* in establishing L‐male mating type, and identify a module that may be associated with other aspects of the distylous syndrome.

In summary, this analysis updates the previously proposed model of SI response in *Turnera*, focusing on how ROS signaling may prevent S‐morph pollen from penetrating self‐styles. We propose that *α‐DOX* ^*S*^ establishes S‐morph female SI response and that *GST8* ensures the L‐morph is capable of penetrating and elongating through the S‐morph's stigma. Future empirical work will focus on the relations *α‐DOX* ^*S*^, and *GST8* have with SI response.

### 3.1 Genes Outside of the *S*‐Locus Confer SC, Supporting YUC6's Role as a Master Regulator

Analysis of mutants has supported the hypothesis that non‐*S*‐locus genes play a crucial role in establishing S‐male mating type. Self‐compatibility was not inherited with the *S*‐locus in the *T. scabra* mutant nor the previously described *T. joelii* T1S2 mutant (Chafe et al. 2015). Thus, SC in these lines arose from mutation(s) to non‐*S*‐locus gene(s), supporting *YUC6's* hypothetical role as a master regulator. The SC *T. scabra* mutant provides the most interesting line of evidence, as SC appears to result from the loss of expression of two genes, *CDCP* and *ILL1*.

CDCP is a hypothetical protein without an assigned function, although it shares 36.6% and 29.49% identity with CDCP; computational analysis confirmed the presence of a cystatin domain in CDCP. The predicted molecular weight of CDCP, 23.5 kDa, suggests that it is part of the type‐II subfamily, PhyCys‐II, which is specific to plants (Balbinott and Margis 2022). PhyCys‐II is capable of inhibiting papain and legumain‐like proteases, but it also shows species‐specific roles (Balbinott and Margis 2022), suggesting that homology alone may not allow prediction of a role for CDCP. CDCP, however, remains a strong candidate for future investigations, as cystatins are highly expressed in reproductive tissues (Zhao et al. 2014), have been shown to affect male fertility (Xin et al. 2016), and regulate local auxin distribution via negative regulation of *YUC* and positive regulation of *PIN* family members during pollen development (Nie et al. 2021). *ILL1* is a putative member of the IAA‐amino hydrolase family. IAA‐amino hydrolases are responsible for maintaining IAA homeostasis as they hydrolyze IAA‐amino acid conjugates resulting in IAA activation or, in the case of IAA‐Asp and IAA‐Glu, degradation (Woodward and Bartel 2005; Hayashi et al. 2021). As additional members of the *ILL* family were identified across our analyses, *ILL1‐4*, was identified as WT‐specific in the T1S2 dataset and upregulated in the SC *T. joelii* dataset. Strict maintenance of auxin homeostasis may be crucial for establishing S‐male mating type.

While SC was seemingly inherited with the *S*‐locus in the SC *T. joelii* line, as all progenies were SC S‐morph or WT L‐morph, the SC mutant and its progeny expressed *YUC6* at WT levels during bud development. Expression of *YUC6* at WT levels suggests that the mutation that confers SC has occurred outside of the *S*‐locus, although inheritance data suggest that the gene is linked to the *S*‐locus. Our co‐expression analysis identified three IAA‐related genes whose expression significantly positively correlated with the presence of SC: *IAA14*, *MES17*, and *CYP79B3*. In *Arabidopsis*, IAA14 reduces response to IAA by negatively regulating *AUXIN RESPONSE FACTORS* (Fukaki et al. 2005). CYP79B3 is part of a different IAA metabolism pathway, which generates indole‐3‐acetaldoxime from TRP, eventually producing either IAA or indole glucosinolates (Mano and Nemoto 2012), though this has only been observed in *Arabidopsis*. MES17 likely negatively regulates auxin signaling via methylation and subsequent inactivation of IAA (Li, Hou, et al. 2008). These results suggest that IAA signaling has been altered in the SC mutant relative to the WT S‐morph and may allude to the insensitivity of YUC6 IAA production likely due to the disruption of transportation. The KEGG pathway for tryptophan metabolism was identified as negatively correlated with the SC phenotype, further suggesting disruption of IAA signaling in the mutant.

YUC6's role as a master regulator was further supported by our co‐expression analysis. *YUC6* was identified as associated with one module in our co‐expression analysis. GO term enrichment analysis suggests the module is involved in auxin biosynthesis. KEGG pathway enrichment analysis of the module identified several pathways associated with auxin biosynthesis, transcription, translation, and post‐transcriptional or translational modifications. The most enriched KEGG pathways were “one carbon pool by folate,” in which C_1_ is metabolized to produce folate (Hanson and Roje 2001), and pyruvate metabolism. In plants, folate has been linked to auxin synthesis and transportation (Gorelova et al. 2019), and pyruvate is a metabolite used during the tryptophan‐dependent auxin biosynthesis pathway (Korasick et al. 2013; Sato et al. 2022). The enrichment of these pathways supports the role of this module in IAA biosynthesis.

Taken together, these results are consistent with YUC6 acting as a master regulator of gene expression via alteration of auxin signaling, which ultimately results in the S‐male mating type. Further investigation into this hypothesis and the aforementioned proteins is warranted.

### 3.2 ROS May Act as a Key Player in SI Response

Previously, an α‐dioxygenase (*α‐DOX* ^*S*^, Tsubulata_027817/KAJ4832334.1) was identified as S‐morph style‐specific (Khosravi et al. 2004). The α‐dioxygenase (α‐DOX) family catalyzes the oxygenation of fatty acids to produce oxylipins (Hamberg et al. 2005). The *α‐DOX* family is found solely in plants, is very small, and rarely undergoes gene duplication (Berthelier et al. 2024). While genomes typically only contain one or two *α‐DOXs* (Berthelier et al. 2024), *T. subulata* 's genome (diploid) contains three *α‐DOXs*, hinting at the possibility of neofunctionalization of one of the family members. Co‐expression analysis suggests *α‐DOX* ^*S*^ plays a role in some aspect of the distylous syndrome as it was identified as part of a module that correlates with female SI, filament elongation, and repression of style elongation. This module was also enriched with genes annotated as peroxisomes, posing the possibility that there are high levels of lipid catabolism/biosynthesis and ROS metabolism in the S‐morph relative to the L‐morph.

The role of the α‐DOX family in oxylipin production and accumulation in conjunction with previous differential expression analysis of the mature pistil, which identified several fatty‐acid‐related genes (Henning et al. 2020), suggests that the S‐morph's pistil contains higher levels of oxylipin than that of the L‐morph's. This may be supported by the *S*‐gene *BAHD*, which establishes female mating type via inactivation of BR (Matzke et al. 2020, 2021).

Decreases in BR concentrations can result in upregulation of jasmonic acid (JA) (Saini et al. 2015). As JA positively regulates *α‐DOX* family members (Hong et al. 2017), S‐morph‐specific expression of α‐DOX may result from increased JA levels in the S‐morph. This suggests that establishing S‐female mating type may rely heavily on BR‐JA antagonistic crosstalk. As oxylipins are byproducts of oxidative damage and help mitigate future oxidative damage (Knieper et al. 2023), there is the possibility that the S‐morph's pistil contains a higher level of ROS relative to the L‐morph. Furthermore, JA has a complicated relationship with ROS, as JA metabolites initiate generation of ROS; while JA positively regulates the transcription of antioxidants (Kolupaev et al. 2023), it is possible that ROS levels show a higher level of regulation in the S‐morph's pistil relative to the L‐morph's.

It should be noted that the relationship between α‐DOX and ROS is complicated as family members can trigger PCD in response to pathogen infection (Vicente et al. 2012; García‐Marcos et al. 2013). As the role of *α‐DOX* ^*S*^ is currently unknown, the possibility remains that S‐morph‐specific expression of α‐DOX^S^ triggers pathogen response pathways in response to self‐pollen, essentially using pathogen defense pathways as a defense for self‐fertilization.

MS analysis heavily suggested Tsubulata_039799 (GST8), a hypothetical GST, may be important for establishing L‐male mating type as representative peptides were identified as DE in nearly every analysis (Table 2). GST8 is a member of the *GST* family class phi. The phi class of *GST* (*GSTF*) is the second largest class and is solely found in plants (Moons 2005). They display a variety of functions, suggesting homology alone cannot determine the hypothetical role of a family member; however, GSTF seems to generally act as peroxidases (Moons 2005; Vaish et al. 2020), helping maintain ROS homeostasis (Tian et al. 2009; Lyall et al. 2020) via transportation of reactive molecules such as oxylipins (Dixon and Edwards 2010).

**FIGURE 5:**
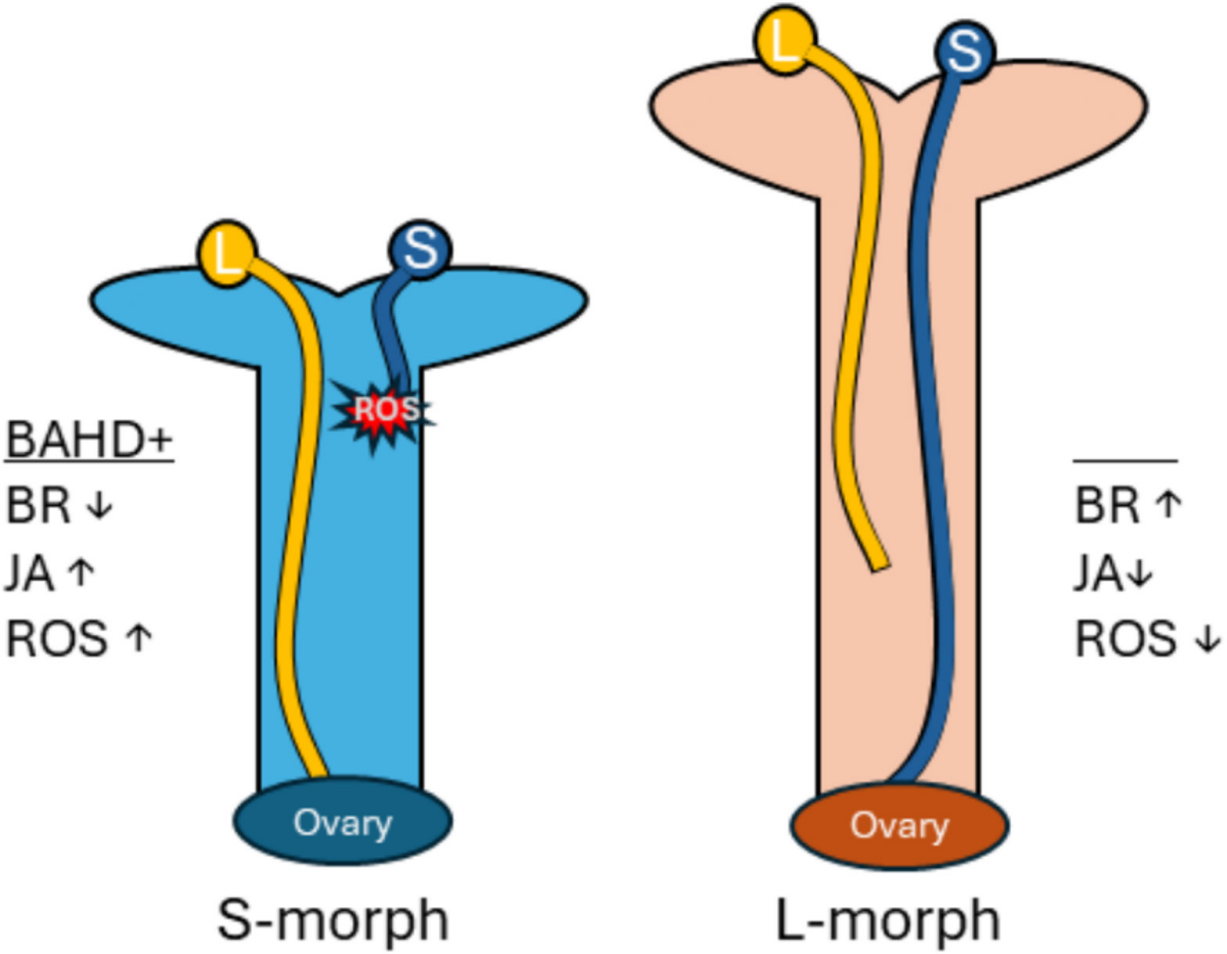
An updated proposal for how SI response is established in *Turnera*. The presence of BAHD in the S‐morph decreases active brassionsteriod (BR) content, causing overall transcriptional changes in the pistil resulting in an increase in jasmonic acid (JA) and reactive oxygen species (ROS). This increase in ROS causes the S‐morph's pollen tube to burst, preventing growth through the style. The L‐morph represents the “default” state of the pistil—high active BR, low JA, and low ROS content. This environment reduces growth rate of the L‐morph's pollen tube and prevents penetration of the tube into the ovary.

Considering the roles of GST family members in ROS homeostasis in addition to JA's ability to trigger ROS production (Mira et al. 2016), the basis of successful fertilization of the S‐morph may be reliant on the pollen's ability to maintain ROS homeostasis (Figure 5), i.e., the L‐morph's pollen is better equipped to survive a high ROS environment. The use of ROS to confer SI is not a novel notion, as increased ROS production in the style during an incompatible cross induces an SI response in *Brassica rapa* (Zhang et al. 2021). Furthermore, in general plant reproduction, the germinating pollen releases an unknown substrate to inhibit peroxidase activity (Breygina and Klimenko 2020); the S‐morph's pollen's inability to penetrate the stigma again is indicative of the inability to maintain ROS homeostasis (Safavian and Shore 2010).

Several ROS‐related proteins were identified across our MS analyses. PAP was only identified in SC *T. joelii*; *PAP* family members' expression is negatively regulated by cytokinin and auxin (Bhadouria and Giri 2022) and are involved in inorganic phosphate (Pi) homeostasis, stress response, general development (Bhadouria and Giri 2022), and ROS homeostasis (Li, Shao, et al. 2008). *AtPAP15* is expressed highly during the maturation of pollen and during germination. Knocking down *AtPAP15* expression results in reduced germination rates, indicating a role in the release of phosphorus reserves during pollen germination (Kuang et al. 2009), while ectopic expression of a soybean *PAP* in *Arabidopsis* resulted in decreased ROS levels and cell death (Li, Shao, et al. 2008). SC *T. joelii* had higher levels of SBT5. *SBT* act as transcriptional regulators via degradation of other transcription factors, and regulate a variety of functions including the induction of PCD via induction of increased ROS production, enhancement of stress response, regulation of methylesterification of cell walls, repression of flowering, in addition to a number of other processes (Schaller et al. 2018; Ali et al. 2022).

In addition to the PhysCys‐II cystatin identified in *T. scabra* , two class PhyCys‐I cystatins were also identified in SC *T. joelii*, and one was identified in the WT SvL analysis. As PhyCys‐I cystatins also inhibit papain‐like proteases, similar to PhyCys‐II, but cannot inhibit legumain‐like proteases unlike PhyCys‐II members. This result may suggest an important role for papain‐like proteases in establishing S‐male mating type. Papain‐like proteases regulate a series of processes including the regulation of PCD in the tapetum and contribution towards the maintenance of ROS homeostasis (Sueldo and van der Hoorn 2017; Liu et al. 2018).

Our co‐expression analysis also identified two *GST‐related* genes, *GPX6* and *NIT1*, as correlated with SC. The GPX and GST families are homologous in function (Dixon and Edwards 2010), making GPX6 co‐occurrence with GST8 of interest. In *Arabidopsis*, NIT1 repairs deaminated glutathione (Niehaus et al. 2019), potentially suggesting high levels of glutathione, a substrate required for *GST* and *GPX* function (Wu et al. 2023), in the SC plant relative to WT S‐morph.

KEGG analysis of the genes whose expression negatively correlates with SC identified enrichment of MAPK signaling. This may pertain to altered ROS response/signaling, as H_2_O_2_ responsive *PATHOGENESIS‐RELATED 1* (*PR1*), which positively responds to high ROS levels (Chaomurilege et al. 2023), and *CATALASE 1*, which negatively regulates ROS levels (Bi et al. 2017) were found in this dataset. Negative correlation of these genes with the SC phenotype may be a response to the positive correlation of *GST* and *GPX* family members with the SC phenotype.

This hypothesis is supported by previous pollen tube work, in which it was determined that PCD, which can be triggered by high ROS levels, may be the root for SI response in the L‐morph (Safavian and Shore 2010). We believe, however, that this may have been a misinterpretation of the data and that PCD is likely causing S‐morph SI response, as S‐morph self‐pollen tubes are disrupted shortly after penetration of the style unlike L‐morph tubes, which remain intact. This is further supported by the differential expression of several PCD and ROS‐related genes, such as purple acid phosphatase, subtilisin‐like serine protease, and the several cystatin family members.

### 3.3 An Improved Model for SI Response in *Turnera*

**FIGURE 6:**
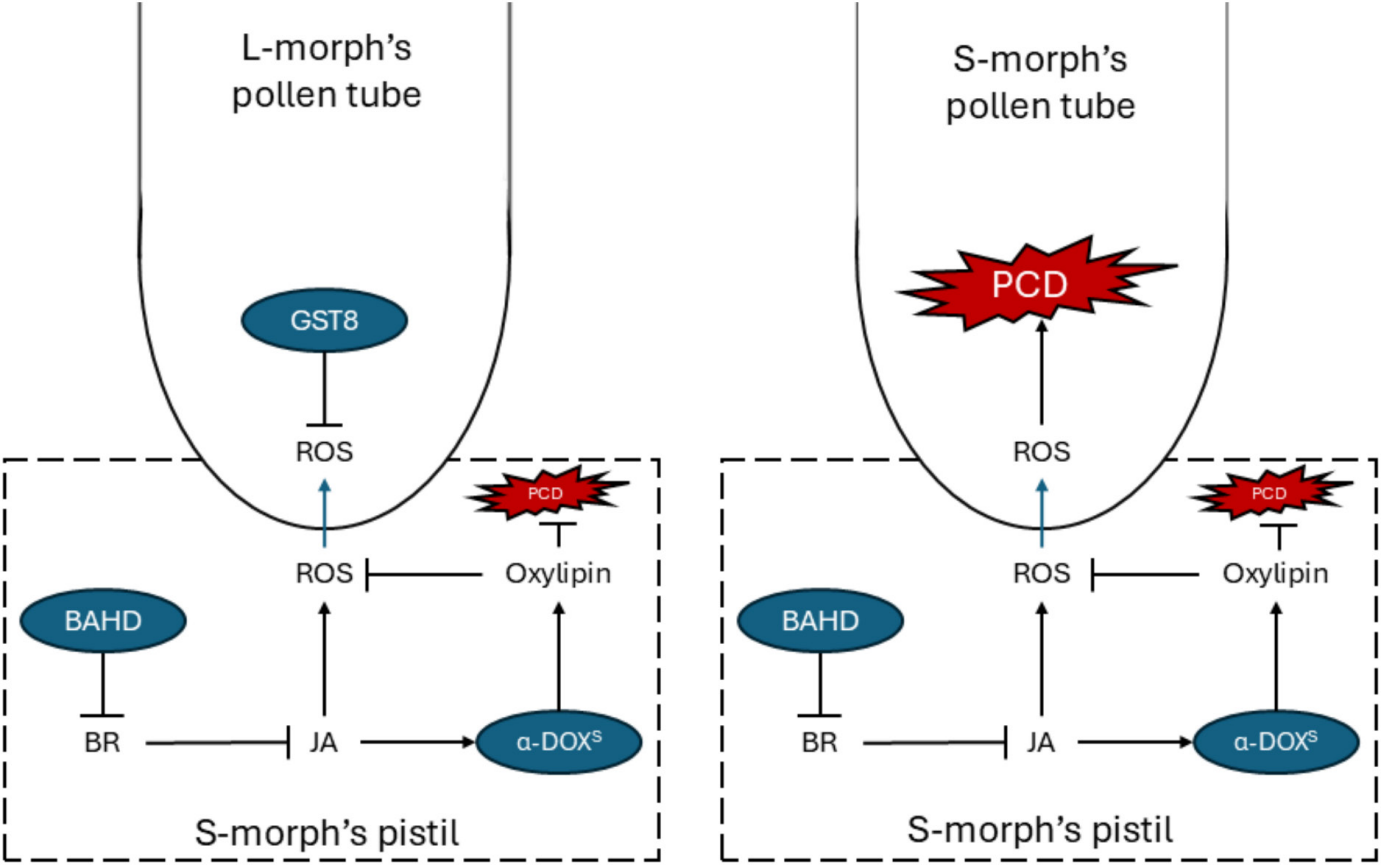
Molecular basis of the proposed model of SI response in *Turnera* during penetration of the L‐morph's (left) or S‐morph's (right) pollen tube*.* The presence of BAHD in the pistil decreases active BR, resulting in an increase in JA. JA promotes the accumulation of ROS and the expression of α‐DOX^S^. α‐DOX^S^ helps maintains a high ROS homeostasis while preventing programmed cell death via the biosynthesis of oxylipins. Secreted ROS is transported into the germinating pollen tube. In the S‐morph's pollen, this increase in ROS content triggers PCD response. In the L‐morph's pollen, the presence of GST8 helps maintain ROS homeostasis ultimately preventing PCD response. BR = brassionsteriods, JA = jasmonic acid, PCD = programmed cell death, ROS = reactive oxygen species. Blue arrow = transportation. Black arrow = repression or promotion.

Here, we expand on a previous *Turnera* SI model to incorporate recent discoveries (Figures 5 and 6). We propose that the inactivation of BR via BAHD disrupts ROS homeostasis resulting in increased levels of ROS in the S‐morph's style relative to the L‐morph's, as supported by the S‐morph‐specific expression of proteins involved in oxylipin production, higher expression of genes related to JA signaling, and the S‐morph‐specific α‐DOX^s^. As ROS homeostasis is important during germination and elongation (Liu et al. 2021; Breygina et al. 2022), higher levels of GST8 expression in the L‐morph's pollen may allow for rapid response to the S‐morph's pistil's hostile environment. The S‐morph's pollen may contain less GST8 due to higher IAA levels; inadequate levels of GST8 in the S‐morph's pollen prevent ROS homeostasis, resulting in premature pollen tube rupturing.

The L‐morph's pollen tube may cease to elongate during incompatible crosses either due to low IAA reserves or the lack of synthesis of proteins related to IAA biosynthesis. The tryptophan‐biosynthesis pathway is seemingly active in the S‐morph's elongating tube, which may cause rapid growth through the L‐morph's much longer style, ensuring sperm delivery. A deeper exploration of the styles' proteome is required to understand the basis of L‐morph SI response.

## 4 Conclusion

This work uses comparative proteomics and transcriptomics to explore the potential basis of male SI response. This analysis identified a member of the GST family that is likely involved in establishing L‐morph male mating type by allowing for pollen tube elongation through a “hostile” environment. Our analysis suggests that female mating type is potentially more complex than previously believed, likely relying on BR‐JA crosstalk to increase ROS levels, as supported by differential expression of ROS responsive proteins GST8, PAP, SBT, PR1, CATALASE 1, and several cystatins in the pollen of SC mutants. The presence of GST8 in the WT L‐morph's pollen likely allows the L‐morph to rapidly respond to high ROS levels, while the lower levels of GST8 in the WT S‐morph's pollen provide an insufficient response resulting in cell death. Overall, this work has identified potential targets for future empirical work exploring the basis of L‐morph male mating type and S‐morph SI response during illegitimate crosses.

## 5 Materials and Methods

### 5.1 X‐Ray Mutagenesis of *T. scabra*

A single plant with ~10 shoots was exposed to X‐rays, with the focus of the X‐ray tube directed at a single shoot. We used a Willick Xplorer 160 CP 3.2‐kW X‐ray system (Willick Engineering, Santa Fe Springs, CA, USA), exposing the plant to the maximum output of the X‐ray machine (160 kVp and 18.9 mA) for 3 min. In this pilot study, we did not expect to generate any mutants, but we were trying to determine if shoots would survive a long X‐ray exposure, in planning for our future mutagenesis studies (Labonne et al. 2010). Remarkably, some weeks after the exposure, we observed fruit being set on a single shoot. We self‐pollinated flowers on shoots of this plant and found that just one shoot became SC, while the others retained their SI. We eventually propagated cuttings from that shoot as well as from SI shoots on the same plant. Cuttings of different shoots are easily rooted in peat pellets by placing the cut end of a shoot bearing an apical meristem in a sterilized peat pellet, sealing it in a magenta box for ~2 weeks until roots emerge through the peat pellet. Shoots are then transplanted into individual pots in standard potting soil.

### 5.2 Plant Material

WT distylous *T. joelii* and *T. subulata* (4×) were growth chamber grown at 25°C 16‐h day and 17°C 8‐h night cycle in Wisconsin, USA. Mutant plants and their WT counterparts were greenhouse grown in Toronto, Canada. All mutant plants show some level of pollen sterility.

The following plants were used:

1. SC *T. joelii* mutant: A WT S‐morph *T. joelii* spontaneously generated a SC branch; this branch was removed from the bush and propagated alongside WT branches from the bush. Upon noticing spontaneous seed set on the branch, subsequent selfing experiments were performed for that branch and the remainder of the individual to confirm that only the branch was SC. SC and SI branches were propagated from the same individual. Grown in Toronto, Canada. *N* = 5 samples from each plant.
2. Offspring of aforementioned mutant: Seed was collected from the SC *T. joelii* to generate a second generation of SC S‐morph. Grown in Toronto, Canada. *N* = 2 samples from offspring.
3. Mutant ESMAN: X‐ray‐generated SC *T. scabra* mutant, see above section for additional details. SC and SI shoots were propagated from the same individual. Grown in Toronto, Canada. *N* = 5 samples from each plant.
4. Mutant T1S2: During the development of a transformation method for *T. joelii* (Chafe et al. 2015), a SC S‐morph plant was generated during the transformation process for unknown reasons. Grown in Toronto, Canada. *N* = 2 samples from T1S2.
5. WT S‐ and L‐morphs of *T. subulata* (tetraploid): Grown in Wisconsin, USA. *N* = 3 plants of each morph, 1 sample per plant.
6. WT S‐ and L‐morphs of *T. joelii*: Grown in Wisconsin, USA. *N* = 3 plants of each morph, 1 sample per plant.

### 5.3 Protein Isolation, MS, and Computational Analysis

The anthers of 2–3 open flowers were collected for each replicate; anthers collected in Canada were frozen and shipped to Wisconsin for protein extraction. Pollen and anthers were vortexed and centrifuged at max speed for 1 min, homogenization buffer was added to the sample, pollen was resuspended via gentle pipetting, and the buffer containing the pollen was moved to a new tube. The following steps were performed in a cold room: 150 μL of cold homogenization buffer (230‐mM sorbitol, 50‐mM Tris–HCl pH 7.5, 10‐mM KC) with inhibitors (for 10 mL of homogenization buffer: 1 pierce protease inhibitor mini tablet 101 [ThermoFisher, WI, USA, catalog: A32953], 1‐mM PMSF, and 2‐mM vanadate) was added to each sample containing anthers and pollen. Tubes were vortexed, centrifuged for 1 min at max speed, and the buffer containing the pollen was carefully moved to a new tube. A plastic pestle was used for homogenization. Samples were centrifuged at full speed for 5 min, and liquid was carefully moved to a new low‐bind tube. The following steps were performed at room temperature: 600 μL 100% methanol, 150 μL 100% chloroform, and 450‐μL water were added to the samples. Samples were vortexed, vented, and centrifuged at full speed for 2 min. The upper phase of samples was discarded. 450 μL methanol was added to the samples, samples were vortexed, centrifuged at full speed for 2 min, and supernatant was poured off. 450 μL of 80% acetone was added to the samples and centrifuged at full speed for 2 min. Supernatant was pipetted off. Pellet was resolubilized in 100–150 μL 8‐M urea/50‐mM ammonium bicarbonate.

BCA assay (ThermoFisher, WI, USA, 485 catalog: #23225) was used for quantification via ThermoFisher Nanodrop 2000. Twenty micrograms of protein was added to an overnight Trypsin/Lys‐C digest following the manufacturer's protocol (Promega, WI, USA catalog: #V5071). Peptides were cleaned using Sep‐Pak C18 cartridges and accompanying protocol (Waters, MA, USA, catalog: WAT054955). Cleaned peptides were dried with a speedvac and resuspended in 20 μL of 0.1% formic acid prior to mass spectrometer analysis.

MS analysis was performed as described in Henning et al. (2024). Proteome Discoverer (v.2) was used for computational analysis following the pipeline as described in Henning et al. (2024). Proteins with a foldchange of < 0.5 or > 1.5 were considered DE (Ting et al. 2009). Raw spectrum files and Proteome Discoverer files have been deposited to the ProteomeXchange Consortium via the PRIDE (Perez‐Riverol et al. 2022) partner repository with the dataset identifiers PXD057029, PXD057031, and PXD058494. Rossmann cores and substrate specificity were predicted using the Rossmann‐toolbox webserver (accessed March 8, 2024; Kamiński et al. 2022). Protein motifs were identified using the web browser InterProScan (accessed March 10, 2024; Quevillon et al. 2005).

### 5.4 RNA Isolation, Sequencing, and Co‐Expression Analysis

Unpublished RNA‐seq data from the mature whole buds of the SC *T. joelii* mutant collected during a previous analysis (Henning et al. 2020), and published RNA‐seq of the mature whole buds from WT *T. joelii* (GenBank PRJNA589060) were analyzed. RNA was isolated from mature buds from the SC *T. joelii* mutant using Concert Plant RNA Reagent and accompanying small‐scale isolation protocol (Invitrogen, USA). RNA was treated with DNase I (Thermo Scientific, Carlsbad, CA, USA) and cleaned using an RNA‐Clean & Concentrator‐5 kit (Zymo Research, Irving, CA, USA). RNA integrity was measured using an RNA Nano 6000 Assay Kit with an Agilent Bioanalyzer 2100 (Agilent Technologies, Santa Clara, CA, USA). Library construction, sequencing (Illumina PE150), quality check, and filtering (removal of reads with a Phred score < 30) were performed by Novogene (Sacramento, CA, USA).

Unpublished data have been uploaded to GenBank (PRJNA1186743). Ubuntu (release 22.19) was used to run all Linux programs, and R‐studio (v.4.3.2; RStudio Team 2020) was used to run all R programs. All programs were run using default parameters unless otherwise stated. Expression was quantified using HISAT2 (v.2.2.1; Pertea et al. 2016), samtools (v1.13; Danecek et al. 2021), and STRINGTie (v.2.2.1; Pertea et al. 2016). RNA was aligned to the *T. subulata* (2×) reference genome (Henning et al. 2023). Undirected co‐expression networks were generated using WGNCA (v1.72‐1; Langfelder and Horvath 2008) and flastcluster (v1.2.3; Langfelder and Horvath 2012). Please refer to the GitHub repository for all values used for WGNCA analysis. The phenotype table provided to WGNCA is provided in Table S5. Scripts used have been uploaded to GitHub (see Data Availability Statement). Unpublished RNA‐seq data have been uploaded to GenBank (PRJNA1186743). Networks were visualized using CytoScape (v3.10.1; Shannon 2003) and uploaded to NDEx (Pillich et al. 2017). GO term and KEGG pathway enrichment were performed using ShinyGO (v.80; Ge et al. 2020) using default settings and the closest *A. thaliana* homologs.

## Author Contributions

Conceptualization: P.M.H. Methodology: all authors. Software: P.M.H. Mutant identification and inheritance analysis: P.D.J.C. Later generational work: H.J.H. Formal analyses: all authors. Resources: P.M.H. and J.S.S. Data curation: P.M.H. Visualization: P.M.H. and J.S.S. Original draft preparation: P.M.H. Review and editing: all authors. All authors have read and agreed to the published version of the manuscript.

## Funding

We would like to thank the National Science Foundation for funding this work (#2208975 awarded to PMH) and an NSERC grant (JSS).

## Conflicts of Interest

The authors declare no conflicts of interest.

## Peer Review

The peer review history for this article is available in the Supporting Information for this article.

## Supporting information


**Figure S1:** Schematic of the SSC S‐morph mutant. SI refers to the normal self‐incompatible branches. SC refers to the mutant self‐compatible branches. The arrow indicates the potential site of which the mutation originated although note the occurrence of one SI branch above three SC branches.
**Figure S2:** Screening for the male mating‐type *S*‐gene *YUC6* in the SC *T. joelii* S‐morph mutant. (SI) refers to samples from self‐incompatible/normal branches; SC refers to self‐compatible branches; (S) S‐morph of *T. joelii*; (L) L‐morph of *T. joelii*; (‐VE) negative control; LAD = 100‐bp ladder.
**Figure S3:** Average expression (FPMK) of the *S*‐genes, *BAHD* (A, B), *SPH1* (C, D), and *YUC6* (E, F), in the SC (L) mutant and WT (R). Comparison of the SC mutant with mature WT buds (A, C, E) and young WT buds (B, D, F).
**Figure S4:** Differentially expressed proteins identified in the four analyses. Diagram generated using DeepVenn (Hulsen 2022).
**Figure S5:** Heatmap representation of module correlation with traits. Number on heatmap represents the *p*‐value. 0 = not correlated, positive values represent positive correlation; negative values represent negative correlation.
**Figure S6:** Enriched GO terms associated with the pink module, which contains *BAHD* and *SPH1*, as determined by ShinyGO.
**Figure S7:** Enriched KEGG pathways associated with the blue module, which contains *YUC6*, as determined by ShinyGO.
**Figure S8:** Modules that correlated with self‐compatibility.
**Figure S9:** Previously identified differentially expressed genes that positively correlated with self‐compatibility.
**Figure S10:** Enriched KEGG pathways positively correlated with self‐compatibly.
**Table S1:** Upregulated and downregulated proteins shared in the two SC *T. joelii* generations.
**Table S2:** Significantly correlated modules.
**Table S3:** NDex IDs for modules' networks.
**Table S4:** pld370125‐sup‐0001‐Supplemental.docx. *Glutathione S‐Transferase* family members whose mRNA levels correlated to self‐compatibility.
**Table S5:** Trait table provided to WGNCA.

**Data S1:** RNA expression data used for WGNCA analysis.

**Data S2:** Proteome Discover results for all mass spec analyses.

**Data S1:** Peer review.

## Data Availability

RNA‐seq data can be accessed at GenBank (GenBank PRJNA1186743). Proteomic data can be accessed at ProteomeXchange (PXD057029, PXD057031, and PXD058494). Scripts and data used for co‐expression and differential gene analyses can be accessed at GitHub (https://github.com/paigehenning/TurneraPollen_2024). Networks can be accessed at NDEx (https://www.ndexbio.org/#/networkset/540329aa‐2807‐11ef‐9621‐005056ae23aa?accesskey=edb7d8997dbd2792fdf084307a3f2cb6b9a90136d68973ae7d971aca9b9f669e); IDs for individual networks are provided in Table S3.
