# Supplementary material for: Comparative Proteomic Analysis of Self‐Compatible Turnera Mutants Suggests Glutathione S‐Transferase 8 Is Involved in Overcoming S‐Morph Female Self‐Incompatibility Response
Source: Plant Direct. 2025 Nov 28;9(12):e70125. doi: 10.1002/pld3.70125 (PMC12661371; doi:10.1002/pld3.70125)
Supplement: Supplementary file 1 — Figure S1: Schematic of the SSC S‐morph mutant. SI refers to the normal self‐incompatible branches. SC refers to the mutant self‐compatible branches. The arrow indicates the potential site of which the mutation originated although note the occurrence of one SI branch above three SC branches. Figure S2: Screening for the male mating‐type S‐gene YUC6 in the SC T. joelii S‐morph mutant. (SI) refers to samples from self‐incompatible/normal branches; SC refers to self‐compatible branches; (S) S‐morph of T. joelii; (L) L‐morph of T. joelii; (‐VE) negative control; LAD = 100‐bp ladder. Figure S3: Average expression (FPMK) of the S‐genes, BAHD (A, B), SPH1 (C, D), and YUC6 (E, F), in the SC (L) mutant and WT (R). Comparison of the SC mutant with mature WT buds (A, C, E) and young WT buds (B, D, F). Figure S4: Differentially expressed proteins identified in the four analyses. Diagram generated using DeepVenn (Hulsen 2022). Figure S5: Heatmap representation of module correlation with traits. Number on heatmap represents the p‐value. 0 = not correlated, positive values represent positive correlation; negative values represent negative correlation. Figure S6: Enriched GO terms associated with the pink module, which contains BAHD and SPH1, as determined by ShinyGO. Figure S7: Enriched KEGG pathways associated with the blue module, which contains YUC6, as determined by ShinyGO. Figure S8: Modules that correlated with self‐compatibility. Figure S9: Previously identified differentially expressed genes that positively correlated with self‐compatibility. Figure S10: Enriched KEGG pathways positively correlated with self‐compatibly. Table S1: Upregulated and downregulated proteins shared in the two SC T. joelii generations. Table S2: Significantly correlated modules. Table S3: NDex IDs for modules' networks. Table S4: pld370125‐sup‐0001‐Supplemental.docx. Glutathione S‐Transferase family members whose mRNA levels correlated to self‐compatibility. Table S5: Trait table provided to WGNC [file PLD3-9-e70125-s003.docx]

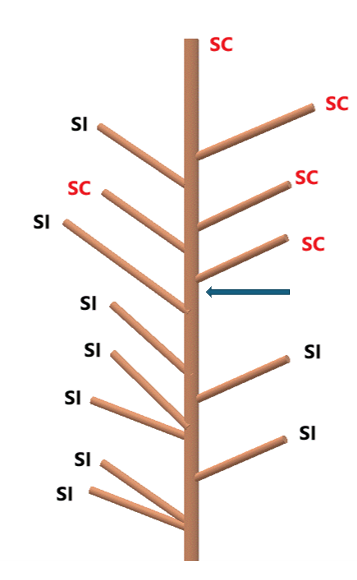


Supplemental figure S1. Schematic of the SSC S-morph mutant. SI refers to the normal self-incompatible branches. SC refers to the mutant self-compatible branches. The arrow indicates the potential site of which the mutation originated although note the occurrence of one SI branch above three SC branches.


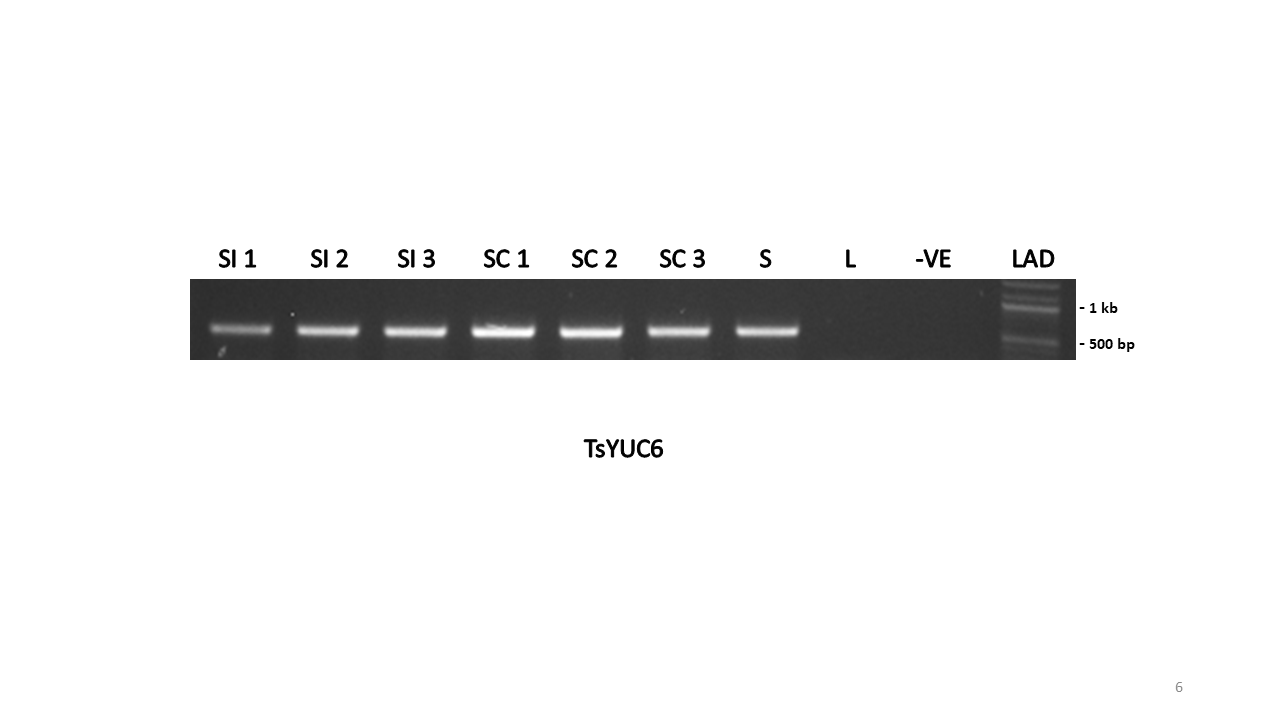


Supplemental figure S2. Screening for the male mating-type *S*-gene *YUC6* in the SC *T. joelii* S-morph mutant. (SI) refers to samples from self-incompatible/normal branches; SC refers to self-compatible branches; (S) S-morph of *T. joelii*; (L) L-morph of *T. joelii*; (-VE) negative control; LAD = 100bp ladder.

|  | **A** | 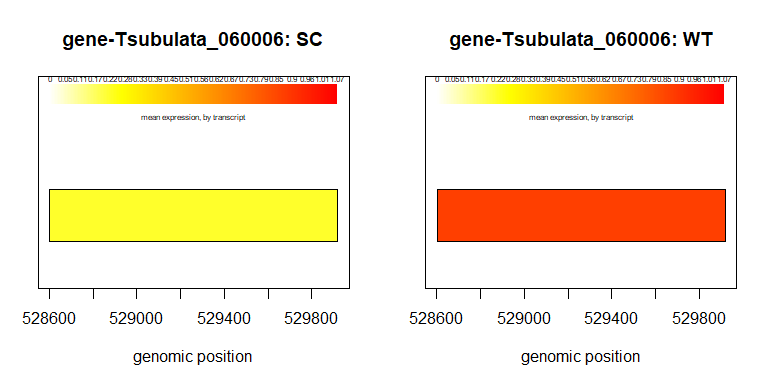 | **B** | 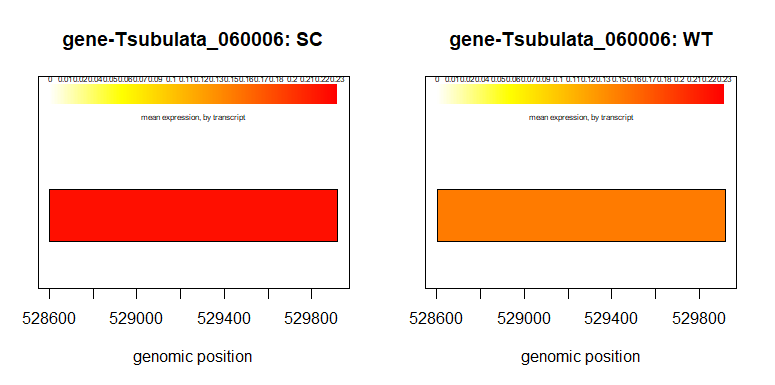 |
| --- | --- | --- | --- | --- |
|  | **C** | 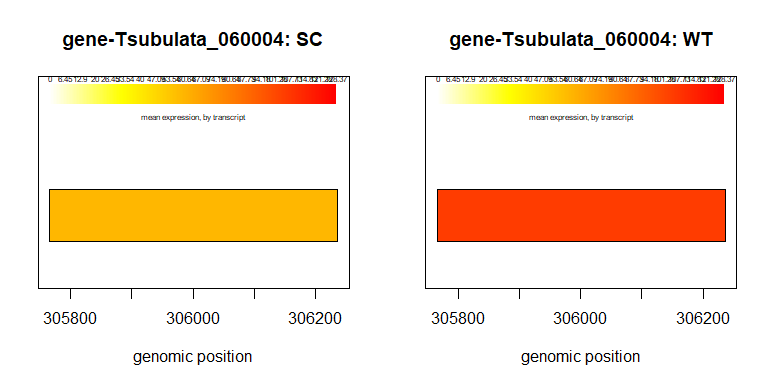 | **D** | 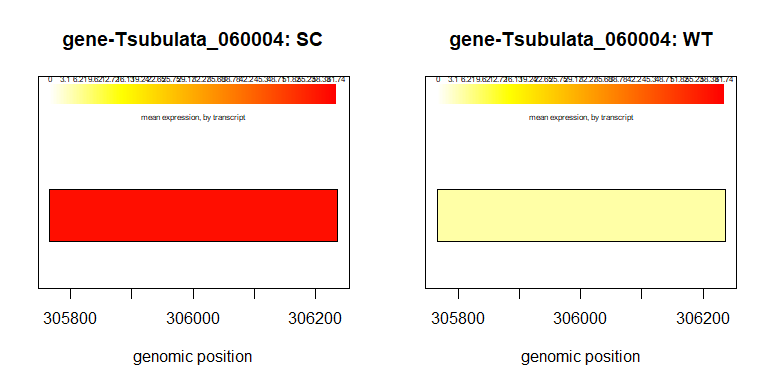 |
|  | **E** | 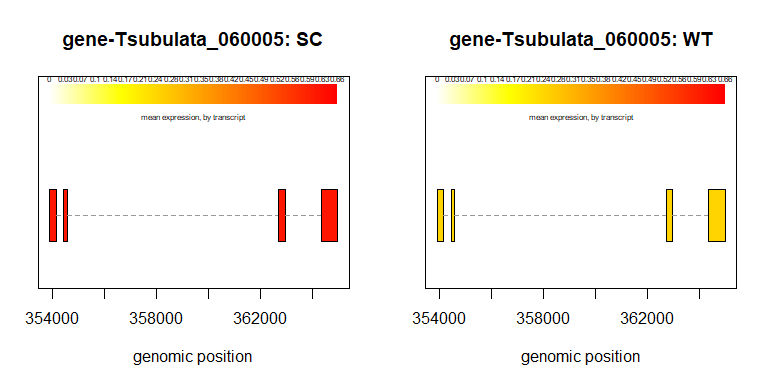 | **F** | 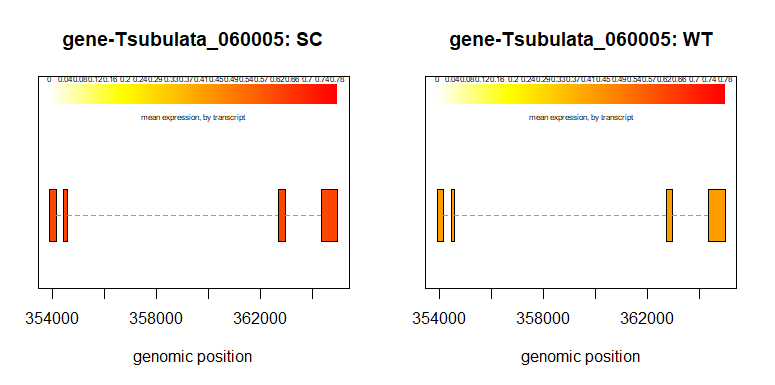 |

Supplemental figure S3. Average expression (FPMK) of the *S*-genes, *BAHD* (A,B), *SPH1* (C,D), and *YUC6* (E,F), in the SC (L) mutant and WT (R). Comparison of the SC mutant with mature WT buds (A,C,E) and young WT buds (B,D,F).


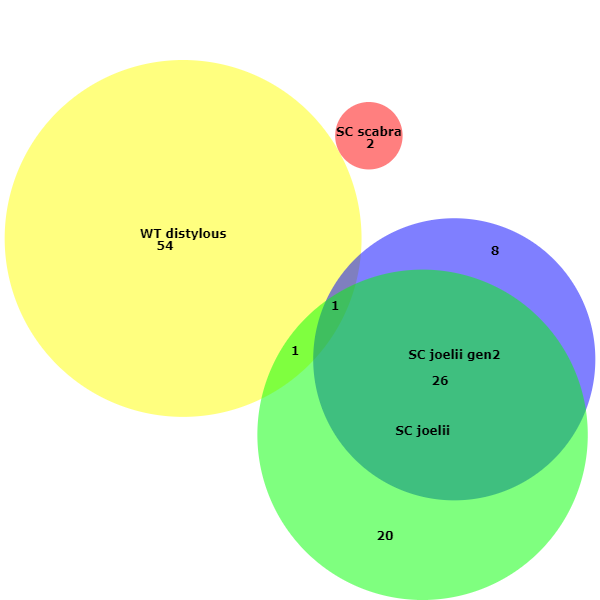


Supplemental figure S4. Differentially expressed proteins identified in the four analyses. Diagram generated using DeepVenn (Hulsen 2022).


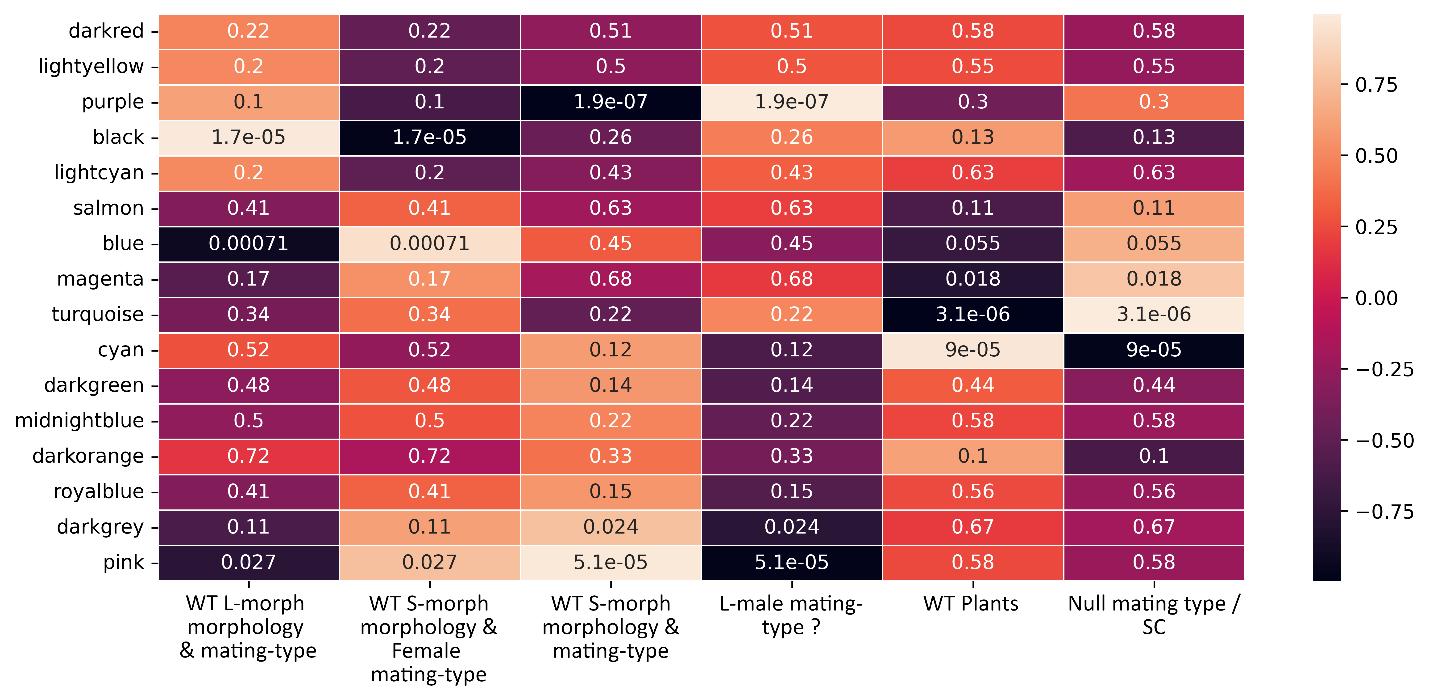


Supplemental figure S5. Heatmap representation of module correlation with traits. Number on heatmap represent the p-value. 0 = not correlated, positive values represent positive correlation, negative values represent negative correlation.


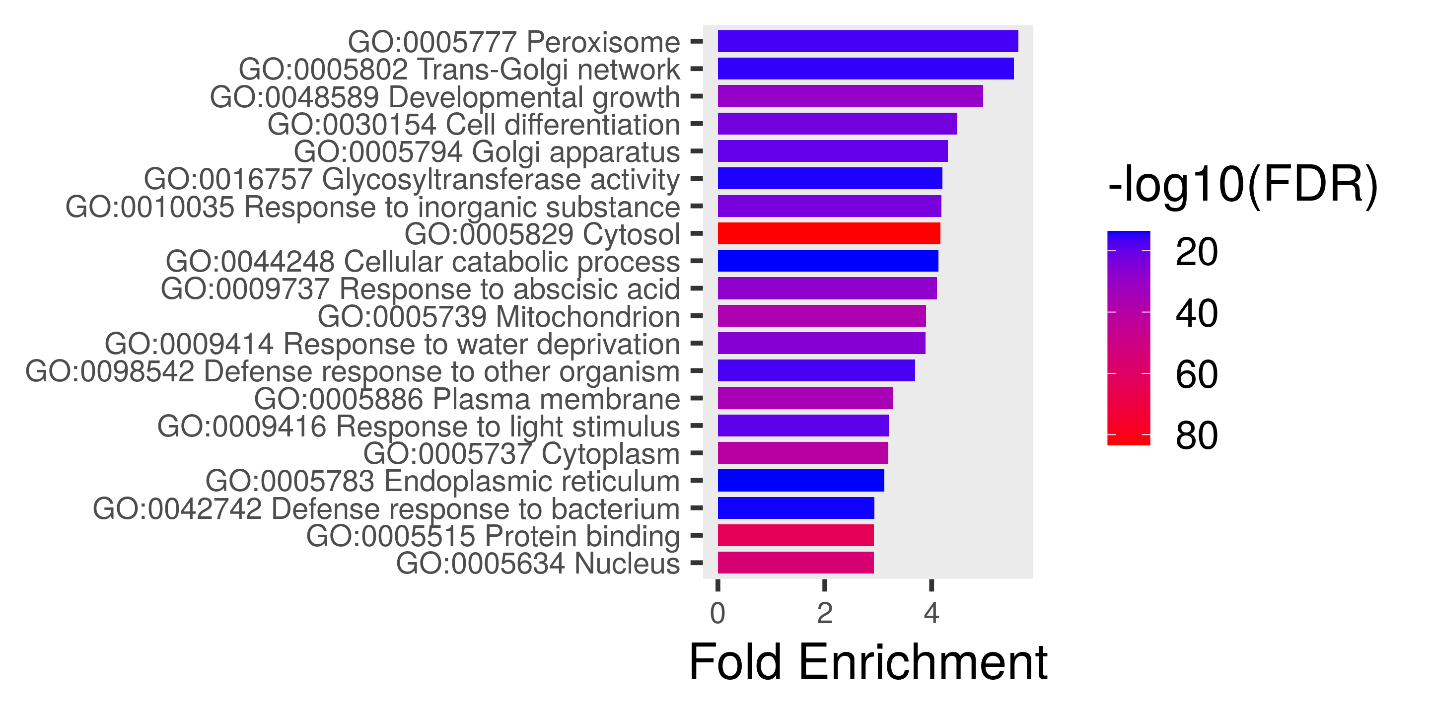


Supplemental figure S6. Enriched GO terms associated with the pink module, which contains *BAHD* and *SPH1*, as determined by ShinyGO.


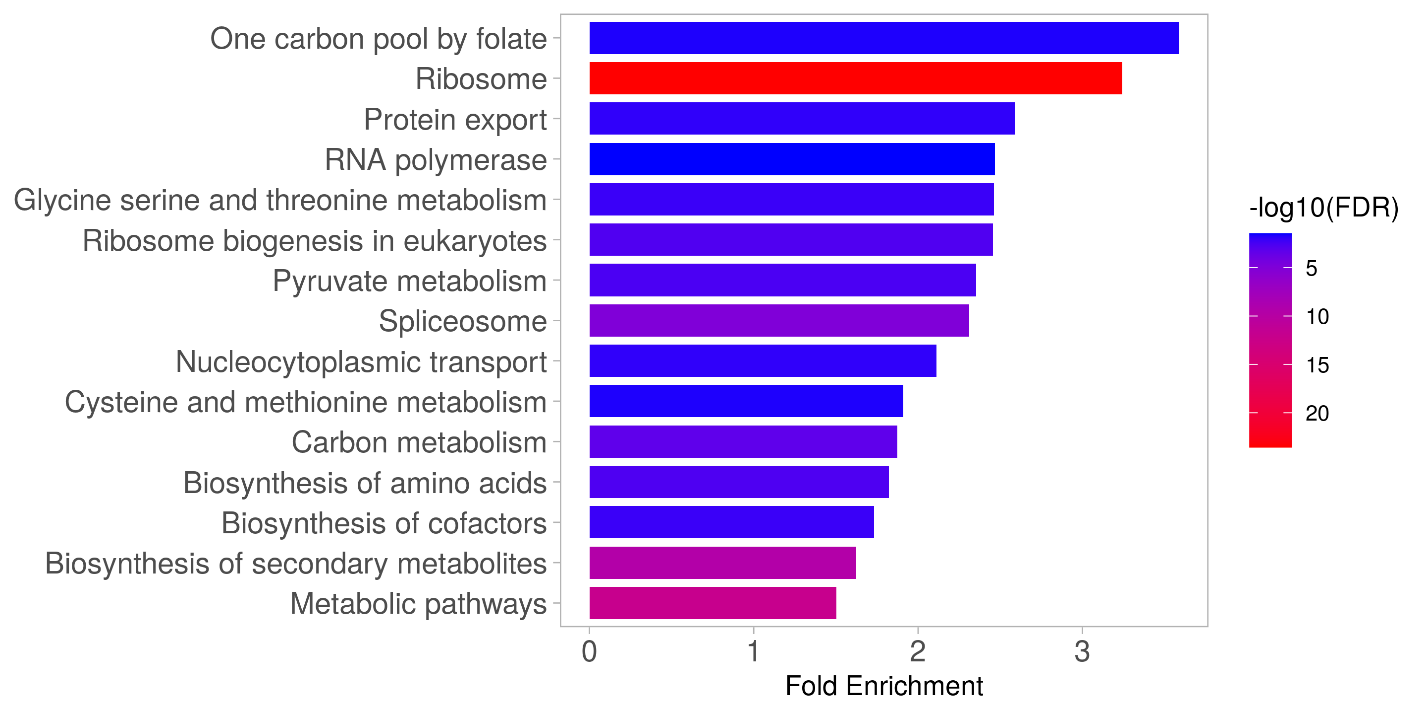


Supplemental figure S7. Enriched KEGG pathways associated with the blue module, which contains *YUC6*, as determined by ShinyGO.


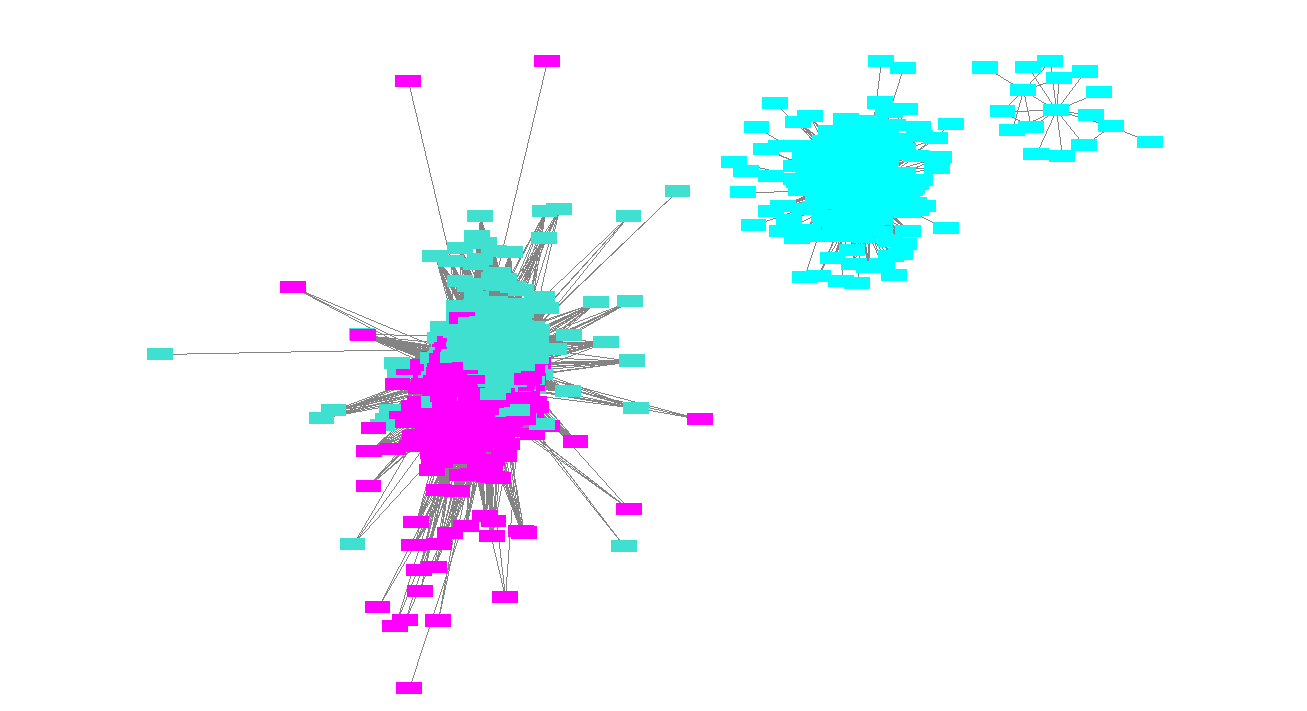


Supplemental figure S8. Modules that correlated with self-compatibility.


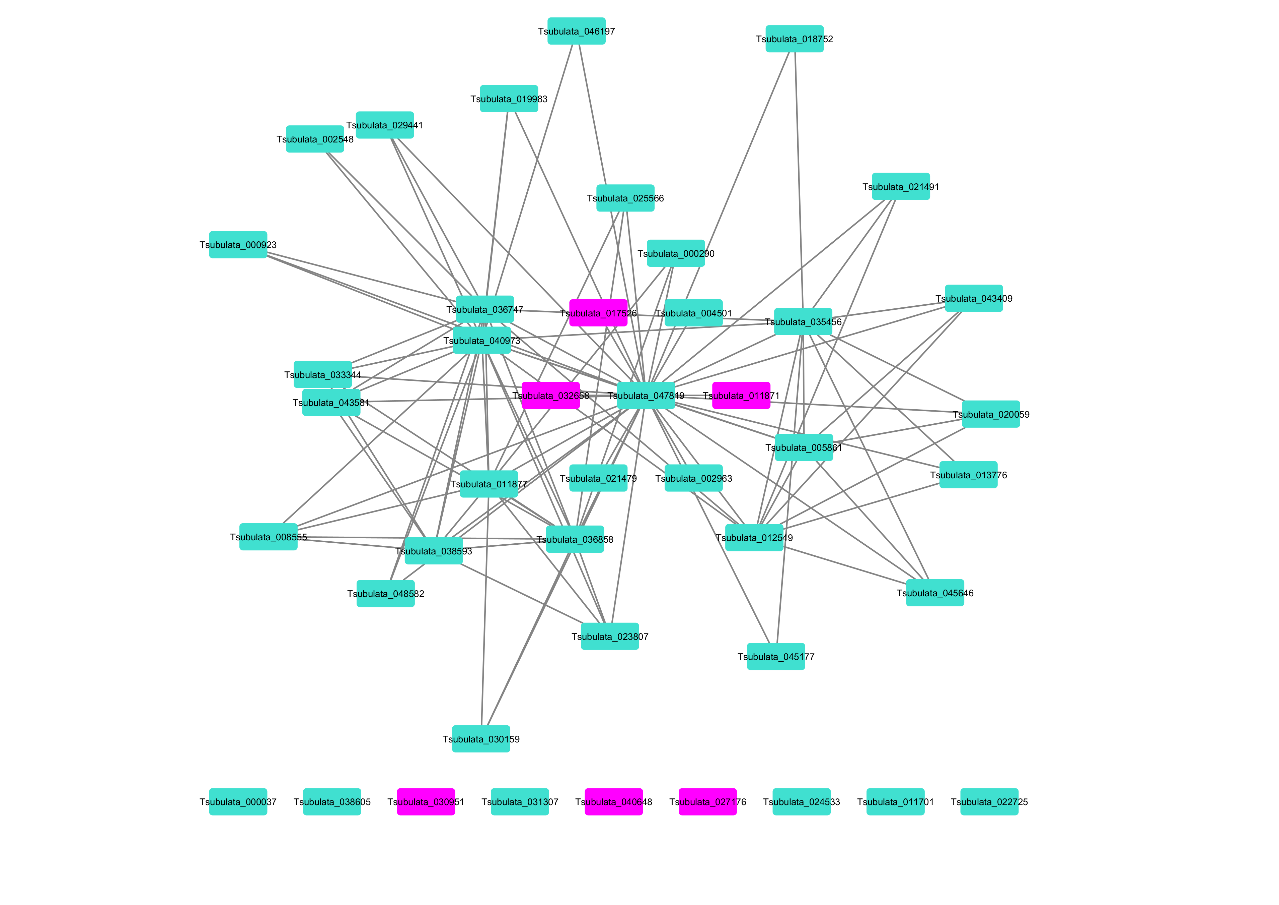


Supplemental figure S9. Previously identified differentially expressed genes that positively correlated with self-compatibility.


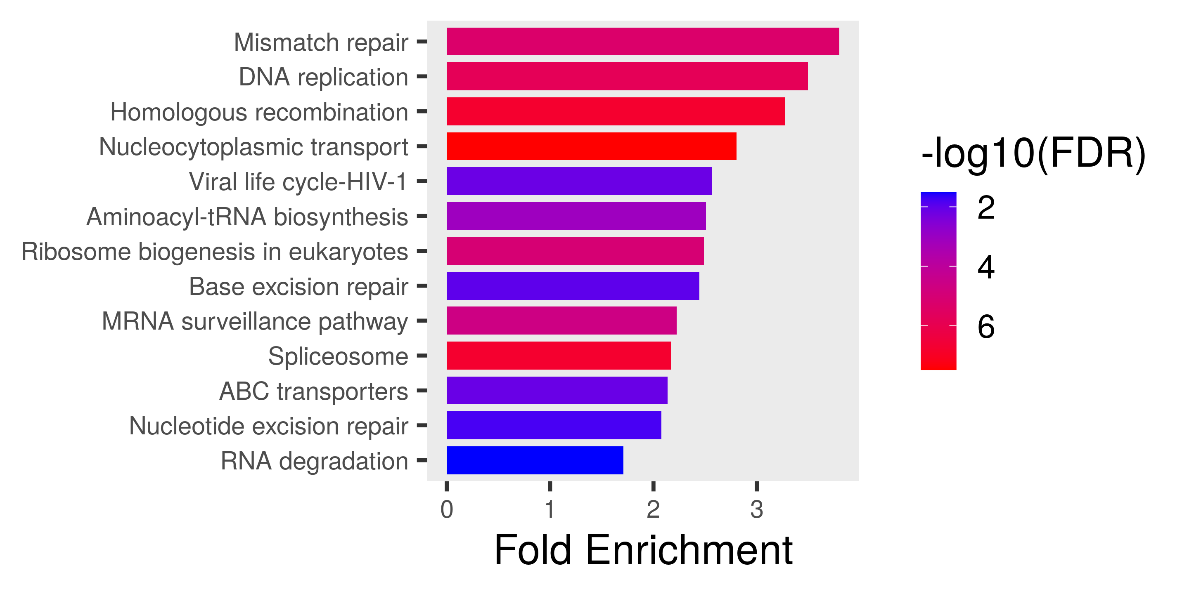


Supplemental figure S10. Enriched KEGG pathways positively correlated with self-compatibly.

Supplemental Table S1. Up- and down-regulated proteins shared in the two SC *T. joelii* generations.

| **Transcript ID** | **Protein ID** | **SC_1_** | **SC_1_&SC_2_** | **Arabidopsis** | **Arabidopsis homolog description (TAIR)** |
| --- | --- | --- | --- | --- | --- |
| Tsubulata_002930 | KAJ4840182.1 | 234.58 | 190.87 | AT2G22930.1 | UDP-Glycosyltransferase superfamily protein |
| Tsubulata_040299 | KAJ4851318.1 | 209.55 | 176.62 | AT4G16500.2 | Cystatin/monellin superfamily protein |
| Tsubulata_001228 | KAJ4825724.1 | 82.14 | 75.173 | AT4G16500.2 | Cystatin/monellin superfamily protein |
| Tsubulata_019629 | KAJ4834260.1 | 53.81 | 54.128 | AT3G08910.1 | DNAJ heat shock family protein |
| Tsubulata_039799 | KAJ4828672.2 | 7.357 | 8.285 | AT1G02920.1 | Encodes glutathione transferase belonging to the phi class of GSTs. |
| Tsubulata_002757 | KAJ4847644.1 | 3.192 | 6.269 | AT2G24050.1 | Encodes a putative eukaryotic translation initiation factor. |
| Tsubulata_004498 | KAJ4823231.1 | 5.62 | 5.048 | AT4G11600.1 | Encodes glutathione peroxidase. Exhibits moderate binding affinity with dinotefuran. |
| Tsubulata_025273 | KAJ4846259.1 | 5.563 | 4.218 | AT1G26230.1 | Encodes a subunit of chloroplasts chaperonins that are involved in mediating the folding of newly synthesized, translocated, or stress-denatured proteins. |
| Tsubulata_039034 | KAJ4830913.1 | 4.145 | 4.153 | AT1G76550.1 | Phosphofructokinase family protein. Target of miRNA sRNA6. |
| Tsubulata_029594 | KAJ4842121.1 | 3.818 | 3.705 | AT2G17420.1 | NADPH-dependent thioredoxin reductase, a major cytosolic isoform. The mRNA is cell-to-cell mobile. |
| Tsubulata_042615 | KAJ4825522.1 | 2.092 | 2.269 | AT1G62660.1 | Glycosyl hydrolases family 32 protein |
| Tsubulata_005313 | KAJ4822851.1 | 2.156 | 2.17 | AT3G04620.1 | Target promoter of the male germline-specific transcription factor DUO1. |
| Tsubulata_015362 | KAJ4846109.1 | 1.615 | 1.836 | AT2G30970.1 | ASPARTATE AMINOTRANSFERASE 1 |
| Tsubulata_033362 | KAJ4841379.1 | 0.455 | 0.377 | AT3G44840.1 | SABATH methyltransferase |
| Tsubulata_013129 | KAJ4829870.1 | 0.369 | 0.368 | AT1G30690.2 | Sec14p-like phosphatidylinositol transfer family protein |
| Tsubulata_026328 | KAJ4824385.1 | 0.305 | 0.362 | AT5G06060.1 | NAD(P)-binding Rossmann-fold superfamily protein |
| Tsubulata_003348 | KAJ4847789.1 | 0.31 | 0.295 | AT2G29940.1 | pleiotropic drug resistance 3 |
| Tsubulata_024898 | KAJ4822694.1 | 0.262 | 0.268 | AT2G37280.3 | Encodes an ATP-binding cassette (ABC) transporter. Expressed in the vascular tissue of primary stem. |
| Tsubulata_005579 | KAJ4823753.1 | 0.257 | 0.266 | AT2G36380.1 | pleiotropic drug resistance 6 |
| Tsubulata_008412 | KAJ4845929.1 | 0.308 | 0.252 | AT1G09740.1 | Adenine nucleotide alpha hydrolases-like superfamily |
|  |  |  |  |  | *Table continued next page* |
| *Table continued* |  |  |  |  |  |
| Tsubulata_022680 | KAJ4825608.1 | 0.267 | 0.233 | AT5G16120.4 | alpha/beta-Hydrolases superfamily protein |
| Tsubulata_002790 | KAJ4835277.1 | 0.09 | 0.166 | AT1G07440.1 | NAD(P)-binding Rossmann-fold superfamily protein |
| Tsubulata_004160 | KAJ4822766.1 | 0.103 | 0.112 | AT1G04010.1 | Encodes a phosphoserine aminotransferase, the second enzyme of the phosphorylation pathway of L-serine biosynthesis. |
| Tsubulata_005577 | KAJ4823751.1 | 0.111 | 0.106 | AT2G36380.1 | pleiotropic drug resistance 6 |
| Tsubulata_033680 | KAJ4847151.1 | 0.085 | 0.086 | AT2G04160.1 | isolated from differential screening of a cDNA library from auxin-treated root culture. encodes a protein similar to subtilisin-like serine protease which is believed to be active outside the plant cell. |

Supplemental Table S2. Significantly correlated modules.

| **Module** | **+/-** | **Trait module correlated with** | **Samples included** |
| --- | --- | --- | --- |
| Purple | +  - | L-male mating-type  General S-morph | L-morph and SC mutant  S-morph only |
| Black | + | General L-morph | L-morph only |
|  | - | S-morphology & female SI | S-morph and SC mutant |
| Blue | + | S-morphology & female SI | S-morph and SC mutant |
|  | - | General L-morph | L-morph only |
| Magenta | + | Self-compatibility | SC mutant only |
|  | - | Self-incompatibility | S- and L-morphs |
| Turquoise | + | Self-compatibility | SC mutant only |
|  | - | Self-incompatibility | S- and L-morphs |
| Cyan | + | Self-incompatibility | S- and L-morphs |
|  | - | Self-compatibility | SC mutant only |
| Dark grey | +  - | General S-morph  L-male mating-type | S-morph only  L-morph and SC mutant |
| Pink | + | S-morphology & female SI | S-morph and SC mutant |
|  | + | S-morph male SI | S-morph only |
|  | - | General L-morph | L-morph only |
|  | - | L-morph male SI | L-morph and SC mutant |

+ = positive correlation. - = negative correlation

Supplementary Table S3. NDex IDs for modules’ networks.

| **Module(s)** | **Description** | **UUID** |
| --- | --- | --- |
| Pink | Correlated with WT S-morph. Contains *S*-genes *BAHD* and *SPH1*. | 62beff8f-485c-11ef-a7fd-005056ae23aa |
| Blue | Correlated with WT S-morph male mating-type. Contains *S*-gene *YUC6*. | 2c548633-485d-11ef-a7fd-005056ae23aa |
| Black, blue, and pink | Three modules correlated with all aspects of the distylous syndrome. | d28e3f6d-4861-11ef-a7fd-005056ae23aa |
| Magenta and turquoise | Positively correlated with SC phenotype. Contains gene of interest *GST8*. | 42f8333a-485f-11ef-a7fd-005056ae23aa |
| Cyan | Negatively correlated with SC phenotype | 0b43c9d6-49cd-11ef-a7fd-005056ae23aa |
| Purple and Dark gray | Two modules that correlated with L-morph male mating-type. Purple positively and dark gray correlated negatively. | afdd68f2-4863-11ef-a7fd-005056ae23aa |

Supplementary Table S4. *GLUTATHIONE S-TRANSFERASE* family members whose mRNA levels correlated to self-compatibility.

| **Name** | **Transcript ID** | **Protein ID** | **DEG (WT Mature stamen)** | **DEP (WT pollen)** |
| --- | --- | --- | --- | --- |
| GST8 | Tsubulata_039799 | KAJ4828672 | No | Down S |
| GST7 | Tsubulata_027213 | KAJ4837327 | No | No |
|  | Tsubulata_004481 | KAJ4823221 | NA | NA |
|  | Tsubulata_017194 | KAJ4850591 | NA | NA |
|  | Tsubulata_001215 | KAJ4825716 | NA | NA |
|  | Tsubulata_039797 | KAJ4828671 | NA | NA |
|  | Tsubulata_539797 | KAK0517591 | NA | NA |
|  | Tsubulata_041635 | KAJ4848139 | NA | NA |

DEG = differentially expressed mRNA (SvL). DEP = differentially expressed peptide (SvL). NA = not previously identified in either tissue.

Supplementary Table S5. Trait table provided to WGNCA

| **Plant** | **Lmorph** | **Smorph** | **Smating** | **Lmating** | **SI** | **SC** |
| --- | --- | --- | --- | --- | --- | --- |
| JSMB3 | 0 | 1 | 1 | 0 | 1 | 0 |
| JSMB2 | 0 | 1 | 1 | 0 | 1 | 0 |
| JSMB1 | 0 | 1 | 1 | 0 | 1 | 0 |
| JSCMB2 | 0 | 1 | 0 | 1 | 0 | 1 |
| JSCMB1 | 0 | 1 | 0 | 1 | 0 | 1 |
| JLMB3 | 1 | 0 | 0 | 1 | 1 | 0 |
| JLMB2 | 1 | 0 | 0 | 1 | 1 | 0 |
| JLMB1 | 1 | 0 | 0 | 1 | 1 | 0 |

1=TRUE. 0 = FALSE. S-mating and L-mating refer to the male mating-type.

**Citations**

Hulsen T. (2022) DeepVenn -- a web application for the creation of area-proportional Venn diagrams using the deep learning framework Tensorflow.js. [online] URL: http://arxiv.org/abs/2210.04597 (accessed 1 March 2024).
