# Supplementary material for: Comparative Proteomic Analysis of Self‐Compatible Turnera Mutants Suggests Glutathione S‐Transferase 8 Is Involved in Overcoming S‐Morph Female Self‐Incompatibility Response
Source: Plant Direct. 2025 Nov 28;9(12):e70125. doi: 10.1002/pld3.70125 (PMC12661371; doi:10.1002/pld3.70125)
Supplement: Supplementary file 4 — Data S1: Peer review. [file PLD3-9-e70125-s001.pdf]

Dear Dr. Mishra,

Thank you for your continued oversight of our manuscript. We sincerely appreciate the peer-review process and its vital role in ensuring the rigor and quality of published research.

While we value constructive feedback, we find it difficult to understand the continued rejection from Reviewer 1. Comment 3 pertains to information that is widely established in the field — q-value universally represents an adjusted p-value. Comments 1, 2, and 5, while potentially valid in nature, appear to involve issues typically addressed during the editorial stage. The remaining comment reiterates a concern that we have already addressed, previously providing multiple references from published studies to support our nomenclature. The comment is merely a matter of semantics, which Reviewer 1 states in their comment.

Given that the second reviewer recommended acceptance after one round of revisions, and that the remaining comments from Reviewer 1 do not materially impact the scientific validity of our work, we are concerned that Reviewer 1 may be unintentionally delaying publication. Should the manuscript be rejected again on similar grounds, we respectfully request that it be re-evaluated by a new reviewer to ensure a fair and unbiased assessment.

Thank you for your time and thoughtful consideration.

Sincerely,

Dr. Paige Henning

Dear Author,
Here are some comments for minor corrections corrections.

1. In results section, each subsections can be give as one or maximum two paragraphs. Avoid several small paragraphs.

>> We appreciate the reviewer's suggestion. However, as this formatting is not a requirement of Plant Direct, and numerous publications in the journal include subsections comprising multiple paragraphs, we have chosen to retain the current structure of the manuscript.

2. Line 165: mean = 0.22 seed, no standard error ?
>>This data was included in the related figure, but now it is also included in the text.

3. Line 215: q-value. Is it adjusted p-value ?
>>q-value universally represents an adjusted p-value

4. Though author gave some references used protein changes were written in expression, to differentiate protein and RNA in paper, I suggest to consider that former can be given as abundance and later can be written in expression. Up-regulated and down-regulated are common for both cases.
>> As noted in our previous response, this point reflects a matter of semantics, as acknowledged at the end of the reviewer's comment. To preserve consistency and readability throughout the manuscript, we have chosen to retain the current terminology.

5. There may be several typo errors. eg. Supplemental figures S7, it should be Supplemental figure S7.
>>Resolved. We are confident that the editorial team will identify any additional typos.
